# The synergistic effect of residues 32T and 550L in the PA protein of H5 subtype avian influenza virus contributes to viral pathogenicity in mice

**DOI:** 10.1371/journal.ppat.1011489

**Published:** 2023-07-03

**Authors:** Hui Yang, Yurui Dong, Ying Bian, Chenzhi Huo, Chuncheng Zhu, Tao Qin, Sujuan Chen, Daxin Peng, Xiufan Liu

**Affiliations:** 1 College of Veterinary Medicine, Yangzhou University, Yangzhou, Jiangsu, China; 2 Jiangsu Co-Innovation Center for the Prevention and Control of Important Animal Infectious Disease and Zoonoses, Yangzhou, Jiangsu, China; 3 Joint International Research Laboratory of Agriculture and Agri-Product Safety, the Ministry of Education of China, Yangzhou University, Yangzhou, Jiangsu, China; 4 Jiangsu Research Centre of Engineering and Technology for Prevention and Control of Poultry Disease, Yangzhou, Jiangsu, China; Washington University in Saint Louis, UNITED STATES

## Abstract

The avian influenza virus (AIV) PA protein contributes to viral replication and pathogenicity; however, its interaction with innate immunity is not well understood. Here, we report that the H5 subtype AIV PA protein strongly suppresses host antiviral defense by interacting with and degrading a key protein in interferon (IFN) signaling, Janus kinase 1 (JAK1). Specifically, the AIV PA protein catalyzes the K48-linked polyubiquitination and degradation of JAK1 at lysine residue 249. Importantly, the AIV PA protein harboring 32T/550L degrades both avian and mammalian JAK1, while the AIV PA protein with residues 32M/550I degrades avian JAK1 only. Furthermore, the residues 32T/550L in PA protein confer optimum polymerase activity and AIV growth in mammalian cells. Notably, the replication and virulence of the AIV PA T32M/L550I mutant are attenuated in infected mice. Collectively, these data reveal an interference role for H5 subtype AIV PA protein in host innate immunity, which can be targeted for the development of specific and effective anti-influenza therapeutics.

## Author summary

The highly pathogenic avian influenza virus (HPAIV) circulates in several countries and causes multiple human infections worldwide. In the present study, we found that two novel residues 32T and 550L in the PA protein of AIV inhibit the IFN-mediated immune response by diminishing JAK1 protein levels and maintaining optimum polymerase activity in mammalian cells. We further revealed that the two amino acids were responsible for the pathogenicity of H5 HPAIV in infected mice. Our study emphasizes the importance of PA protein in the increased pathogenicity of HPAIV and provides important insights for understanding the molecular mechanisms of virus-host interactions, which are critical for developing control strategies for HPAIV.

## Introduction

An increasing number of highly pathogenic avian influenza viruses (HPAIV), including the H5 and H7 subtypes, have acquired the ability to cross the species barrier to infect and kill humans, continually posing a serious threat to public health [1–3]. In 1997, human infection with the H5N1 virus in Hong Kong caused six deaths out of 18 infected individuals [1]. Over the years, H5N1 has become a global epidemic with a mortality of 50–60% [2]. Since H7N9 emerged in humans, at least five epidemics have been recorded in China from 2013–2017, with both geographical distribution and genetic diversity expanding [3–5]. In 2014, another novel avian subtype H5N8 virus emerged in Asia and Europe, causing widespread death in poultry and wild birds [6,7]. In February 2021, the first case of a human infected with a novel H5N8 virus was reported in Russia [8]. In addition, H5N2 or H5N6 reassortants circulate in poultry flocks and occasionally infect humans [9–12]. Currently, H5N1 clade 2.3.4.4b viruses have spread to many countries and have infected mammalian species [13,14]. Therefore, this is alarming that the pandemic potential of H5 AIVs in humans should not be disregarded.

The interferon (IFN) response constitutes an essential part of host defenses that restrict viral infections. IFNs are the primary antiviral cytokines produced by infected cells. The engagement of type I IFNs and their cell surface receptors activates JAK-signal transducer and activator of transcription (STAT) signaling, which promotes the transcription of an extended array of IFN-stimulated genes (ISGs) to exert antiviral activity [15,16]. The production of ISGs is a battle between the host and the virus, which profoundly affects the subsequent replication and spread of the virus. The ubiquitin-proteasome system (UPS) is a key cellular process involved in various pathways, including the virus-dependent innate immune response. TRIM25 [17–19], TRIM14 [20], TRIM41 [21], TRIM32 [22], and other UPS factors are also involved in the antiviral pathway during influenza A virus (IAV) infection, either by amplifying immune responses or by targeting viral proteins [23]. IAVs have evolved to inhibit the host immune response by hijacking host ubiquitin-dependent signaling pathways or by interfering with the cellular ubiquitination machinery to promote viral replication and pathogenesis. Several viral proteins have been found to interfere with ISG production through the UPS mechanism. For example, the viral NS1 protein, the main IAV virulence factor, acts by interacting with RIG-I or UPS proteins such as TRIM25, thereby interfering with ISGs activation [18,24]. Moreover, IAV HA and PB2 expression mediates ubiquitination-dependent proteasomal degradation of IFNAR1 and JAK1, respectively, to attenuate the IFN-induced antiviral signaling pathway [25,26]. However, the antagonistic immune response involving the host ubiquitin system hijacked by IAV PA protein has not been investigated.

The IAV RNA-dependent RNA polymerase complex, which is composed of PB2, PB1, and PA subunits, is essential for viral genome transcription and replication and determines the evolution of adaptive viral changes to overcome host restrictions [27]. PB2-E627K is a well-characterized human adaptation mutation associated with enhanced polymerase activity, virus replication, transmissibility, and virulence of AIV in mammals [28,29]. Different mutations in PB2 have been identified that contribute to the adaptation of influenza viruses to mammalian hosts, including PB2-701N [30], PB2-591R/K [31], PB2-271A [32], PB2-158G [33], PB2-147T/339T/588T [34] and PB2-283M/526R [35,36]. PB1 encodes amino acids that affect polymerase activity and/or transmissibility of H5N1 influenza viruses in mammals, including PB1-K577E [37] and PB1-612K [38]. PA encodes an endonuclease that cleaves the cap structure and subsequent nucleotides from cellular mRNA to generate primers for influenza virus transcription. A recent study reported that low polymerase activity due to PA in the trimeric polymerase complex can drive PB2-E627K substitution in human cells, indicating a functional association between PB2 and PA during AIV adaptation in mammals [39]. The PA protein contains several residues associated with the pathogenicity of AIV in mammals, including PA-343S [40], PA-353R [41], and PA-356R [42]. Although a few cellular factors have been shown to bind PA and affect polymerase function, the exact underlying molecular mechanisms are largely unclear. In this study, we reported that the synergistic effect of residues 32T and 550L in the PA protein of H5 AIV is involved in the regulation of the antiviral innate immune response, which contributes to the pathogenicity of AIV in mice.

## Results

### AIV PA protein promotes polyubiquitination degradation of mammalian JAK1

In a recent study, we showed that the IAV PB2 proteins mediate the degradation of human JAK1 to benefit its replication [26]. To further elucidate whether the other viral proteins contribute to the degradation of human JAK1, we engineered nine 3×Flag-tagged eukaryotic expression plasmids encoding the individual viral proteins (PB1, PA, HA, NP, NA, M1, M2, NS1, and NS2) from A/Puerto Rico/8/34 (H1N1, PR8) and A/goose/Eastern China/CZ/2013 (H5N8, CZ). We then screened for their ability to regulate the expression of human JAK1. The results showed that M1, NS1, and M2 proteins reduced the expression of human JAK1, whereas PB1, HA, NP, NA, and NS2 proteins did not influence human JAK1 protein level (S1 Fig). Interestingly, PA-CZ, but not PA-PR8, downregulated human JAK1 protein. Therefore, we explored how PA from CZ negatively regulates the expression of human JAK1 and the subsequent effect on viral pathogenicity.

**Fig 1:**
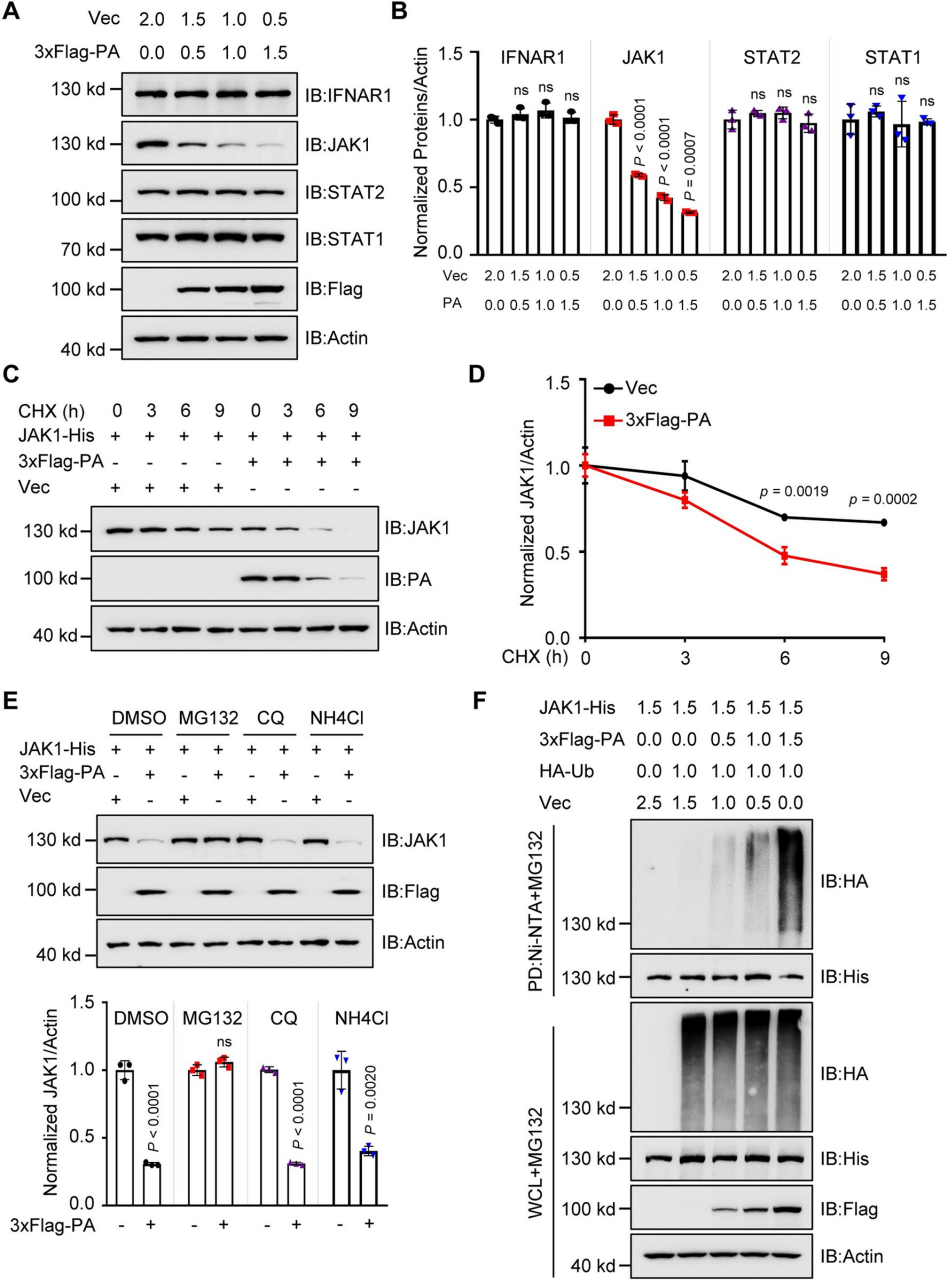
AIV PA protein promotes the degradation of JAK1. (**A**) Immunoblots of HEK293T cells were transfected with indicated plasmids, and the amount of each plasmid was indicated above the lanes (in micrograms). **(B)** Quantification of protein expression on immunoblots (A) and normalized with actin (n  =  3). **(C)** Immunoblots of HEK293T cells were transfected with indicated plasmids and treated with CHX. **(D)** Quantification of protein expression on immunoblots (C) and normalized with actin (n  =  3). **(E)** Immunoblots of HEK293T cells were transfected with indicated plasmids and treated with DMSO, MG132, chloroquine (CQ), or NH4Cl (upper). Quantification of protein expression on the immunoblots and normalized with actin (n  =  3) (lower). **(F)** Ni-NTA pull-down analysis of the ubiquitination of JAK1 in HEK293T cells transfected with indicated plasmids, and the amount of each plasmid was indicated above the lanes (in micrograms), and treated with MG132. WCL, whole-cell lysates. Data are representative of three independent experiments. Data are presented as the mean ± SD. Statistical significance in **B**, **D**, and **E** was determined using the unpaired two-tailed Student’s *t*-test. ^ns^ *P*>0.05.

It was found that PA-CZ downregulated endogenous JAK1 protein but not IFNAR1, STAT1, or STAT2 in HEK293T cells (Fig 1A and 1B). To solidify the effect of PA on the JAK1 expression, we generated a Flag-GST expression plasmid and found that Flag-GST had no effect on the expression of human JAK1; however, Flag-PA significantly reduced the expression of human JAK1, Flag-PB2 was included as a positive control (S2A Fig). Furthermore, when HEK293T cells were transfected with JAK1-His or Flag-STAT1, along with increasing amounts of PA-CZ protein, we observed dose-dependent reductions in the levels of JAK1-His (S2B Fig) but not STAT1 (S2C Fig). We also found that the PA-CZ protein did not affect the expression of eGFP protein (S2D Fig) and Renilla luciferase (Rluc) levels (S2E Fig). PA expression did not alter the mRNA level of JAK1 (S2F Fig). In addition, we used cycloheximide (CHX) to block new protein synthesis and found that the inhibition of JAK1 expression induced by CHX was stronger in the presence of PA (Fig 1C and 1D), indicating that PA-CZ protein specifically decreased the stability of human JAK1 protein. Moreover, the presence of PB2 but not PB1 exhibits an additive effect with PA on the degradation of JAK1 protein (S2G Fig). Meanwhile, the degradation of the JAK1 was also confirmed in the CZ virus-infected HEK293T cells (S2H Fig). However, the expression of JAK1 was not affected by IFNβ treatment (S2I Fig), which is consistent with a previous report [43].

The ubiquitin-proteasome and autophagy-lysosome pathways are two systems that control protein degradation in eukaryotic cells. We found that PA-mediated human JAK1 degradation was mostly restored by treatment with the proteasome inhibitor MG132, but not with the lysosome inhibitor ammonium chloride (NH4Cl) or chloroquine (CQ) (Fig 1E). Next, we determined whether PA promotes the ubiquitination of JAK1. As anticipated, PA-CZ potentiated JAK1 ubiquitination in a dose-dependent manner (Fig 1F). Different polyubiquitination processes, including K48-, K63-, K11-, and K27-linked ubiquitination, have been implicated in deliberately regulating protein fate. Overexpression of PA markedly promoted K48-linked ubiquitination of JAK1 but had no significant effect on the ubiquitination of JAK1 with other linkages (S3 Fig). These data suggest that PA promotes K48-linked ubiquitination and JAK1 degradation through the proteasomal pathway.

### The lysine 249 residue of JAK1 is critical for PA-mediated degradation

**Fig 2:**
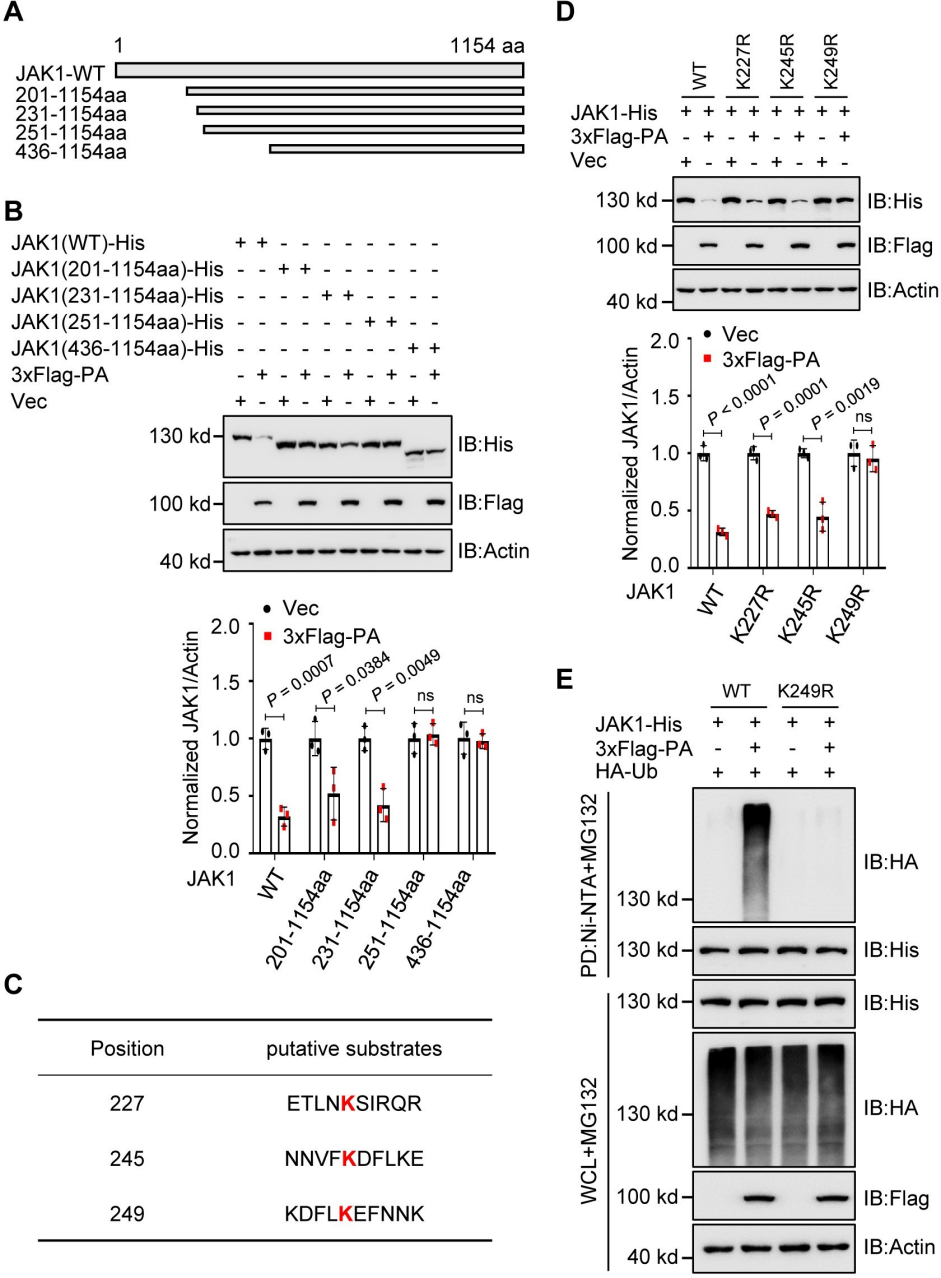
AIV PA protein mediates JAK1 degradation at residue 249K. **(A)** Schematic representation of the deletion mutants of JAK1. **(B)** Immunoblots of HEK293T cells transfected with JAK1 or its deletion mutants and PA plasmids (upper). Intensities of the bands on immunoblots from three independent experiments were quantified and normalized with actin (lower). **(C)** Ubiquitination modification online prediction of JAK1 using CPLM 1.0. **(D)** Immunoblots of HEK293T cells transfected with JAK1 or its mutants and PA plasmids (upper). Intensities of the bands on immunoblots from three independent experiments were quantified and normalized with actin (lower). **(E)** Ni-NTA pull-down analysis of the ubiquitination of JAK1 in HEK293T cells transfected with JAK1 or its mutants, HA-Ub and PA plasmids, and treated with MG132. WCL, whole-cell lysates. Data are representative of three independent experiments. Data are presented as means ± SD and statistical significance was determined by unpaired two-tailed Student’s *t*-test in **B** and **D**. ^ns^ *P* <0.05.

To identify the key residues in JAK1 that are targeted by PA for ubiquitination and degradation, we first designed and constructed truncated plasmids (Fig 2A). JAK1-WT, JAK1-201-1154aa, and JAK1-231-1154aa, but not JAK1-251-1154aa and JAK1-436-1154aa, were extensively degraded by PA (Fig 2B). Next, we mutated a series of JAK1 lysines (Fig 2C) and examined their effects on PA-mediated JAK1 degradation. As shown in Fig 2D, only the mutation of lysine 249 to arginine (K249R) abolished the PA-mediated degradation of JAK1 (Fig 2D). To further dissect the ubiquitination of JAK1 at the K249 residue, JAK1-WT was strongly ubiquitinated when HA-tagged wild-type (WT) ubiquitin was expressed, whereas ubiquitination of JAK1 (K249R) was significantly reduced (Fig 2E). These data indicated that the lysine 249 residue of JAK1 is critical for PA-mediated degradation.

**Fig 3:**
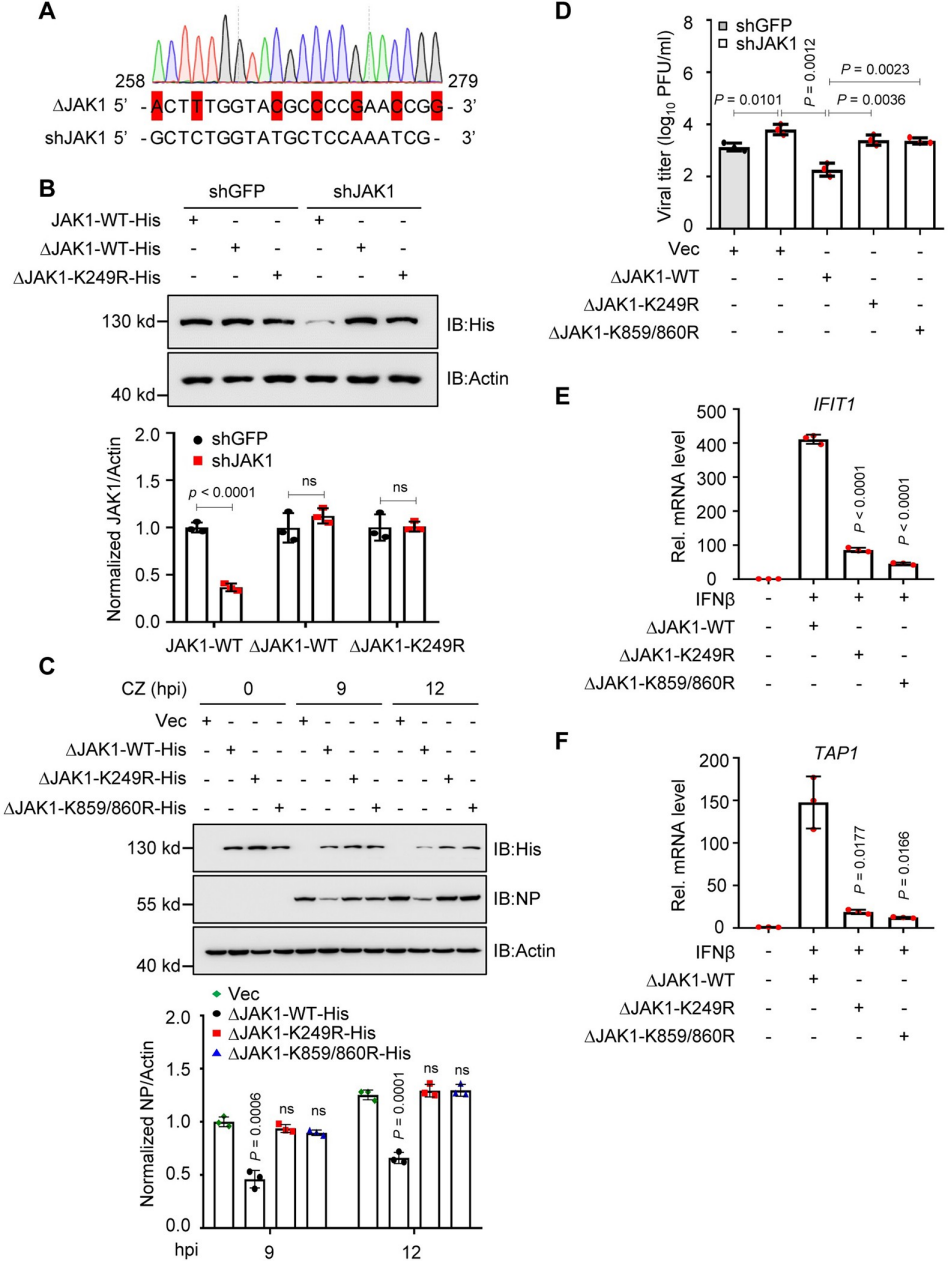
The Lysine 249 residue is critical for JAK1-mediated antiviral activity. **(A)** JAK1 was replaced with an shRNA off-target JAK1 mutant (ΔJAK1) with a 7-nucleotide nonsense mutation in the target sequence of the shJAK1 plasmid. **(B)** Immunoblots of stable JAK1 knockdown HEK293T cells transfected with JAK1-His, ΔJAK1-His, or ΔJAK1-K249R-His plasmid (upper). Intensities of the bands on immunoblots from three independent experiments were quantified and normalized with actin (lower). **(C)** Immunoblots of shJAK1 HEK293T cells transfected with ΔJAK1-WT, ΔJAK1-K249R, or ΔJAK1-K859/860R plasmid and infected with CZ virus (upper). Intensities of the bands on immunoblots from three independent experiments were quantified and normalized with actin (lower). hpi, h post-infection. **(D)** Viral titers in shGFP or shJAK1 HEK293T cells transfected with ΔJAK1-WT, ΔJAK1-K249R, or ΔJAK1-K859/860R plasmid and infected with CZ virus (n = 3). **(E, F)** qPCR analysis of *IFIT1* **(E)** and *TAP1* **(F)** mRNA in shJAK1 HEK293T cells transfected with ΔJAK1-WT, ΔJAK1-K249R, or ΔJAK1-K859/860R plasmid and treated with IFNβ. Data are presented as the mean ± SD and are representative of three independent experiments. Statistical significance in **B**–**F** was determined using the unpaired two-tailed Student’s *t*-test. ^ns^ *P* >0.05.

To further understand the role of JAK1 (K249R) during IAV infection and eliminate the effect of endogenous JAK1 on viral replication, we established a JAK1 shRNA-knockdown (shJAK1) cell line and constructed a mutant JAK1 expression plasmid (ΔJAK1) that contains 7-nucleotide nonsense mutations in the shJAK1 target sequences to restore the JAK1 expression in shJAK1 cells (Fig 3A and 3B). In the CZ virus-infected shJAK1 cells, restoration of JAK1 WT significantly decreased viral NP expression, whereas overexpression of JAK1 K249R, as well as JAK1 K859/860R, had no impact on the viral NP expression (Fig 3C), suggesting that JAK1 WT restoration, but not JAK1 K249R or JAK1 K859/860R overexpression inhibited viral replication which was confirmed by the titers in the supernatant of the cells (Fig 3D). Next, we investigated the effect of JAK1 K249R on the IFN-induced expression of ISGs (interferon-induced protein with tetratricopeptide repeats 1, *IFIT1*, and *TAP1*). The qRT-PCR results indicate that the *IFIT1* and *TAP1* mRNA levels were dramatically decreased in the presence of JAK1 K249R or JAK1 K859/860R overexpression in the shJAK1 cells by comparing with that of JAK1 WT restoration (Fig 3E and 3F), suggesting that the mutation of K249R at JAK1 alleviates the IFNβ-mediated innate immune response. Collectively, our results indicate that PA-mediated JAK1 ubiquitination degradation at K249 residues is critical for efficient AIV replication.

### Synergistic effect of PA residues 32T and 550L on the degradation of mammalian JAK1

**Fig 4:**
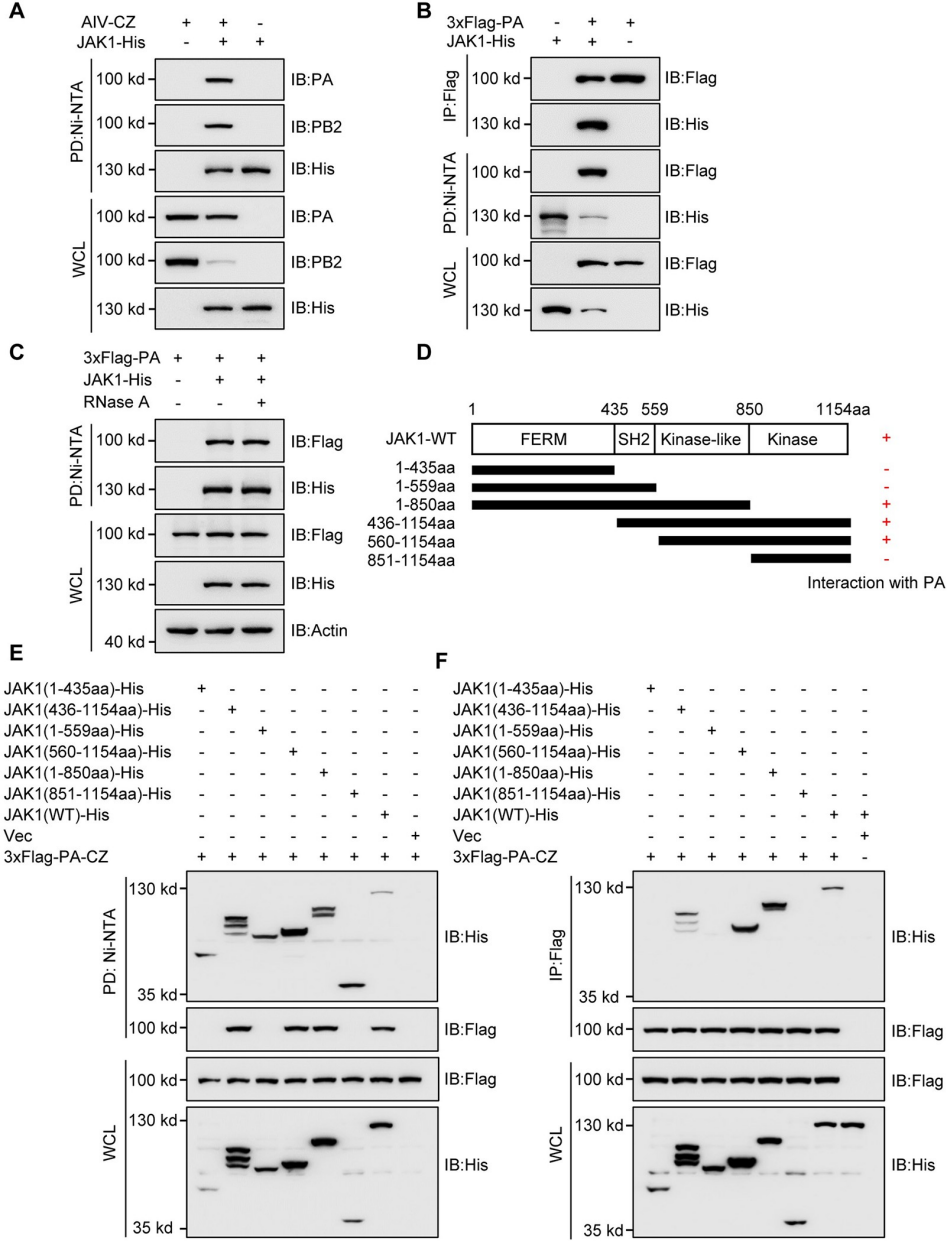
Interaction between JAK1 and AIV PA protein. **(A)** Ni-NTA pull-down analysis of the interaction of PA or PB2 with JAK1 in HEK293T cells transfected with JAK1 plasmids and infected with CZ. **(B)** Co-IP and Ni-NTA pull-down analysis of the interaction of PA with JAK1 in HEK293T cells transfected PA and JAK1 plasmids. (C) Ni-NTA pull-down analysis of the interaction of PA and JAK1 in HEK293T cells transfected with PA and JAK1 plasmids and treated with RNase A. **(D)** Schematic representation of the deletion mutants of JAK1. **(E, F)** Ni-NTA pull-down **(E)** and Co-IP analysis **(F)** of the interaction of PA with JAK1 and its truncation mutants in HEK293T cells. WCL, whole-cell lysates. Data are representative of two independent experiments.

Since a previous study has shown that JAK1 interacts with the IAV PB2 protein [26], we determined whether JAK1 is associated with PA by Nickel (Ni)-NTA pull-down and immunoprecipitation (IP) experiments. The results showed that PA and PB2 proteins could be pulled down simultaneously by JAK1-His under CZ virus infection in HEK293T cells (Fig 4A). Further, IP 3×Flag-PA brought down His-tagged JAK1, and 3×Flag-PA was pulled down with His-tagged JAK1 (Fig 4B), indicating that JAK1 interacts with the PA protein. As the PA protein is associated with RNA, we further performed the JAK1-PA pull down assay in presence of RNase A. The result showed that the presence of RNase A did not affect the JAK1-PA interaction (Fig 4C). To map the region of JAK1 responsible for interaction with PA, we designed and constructed truncated plasmids (Fig 4D). Deletion of the kinase-like domain (amino acids 560–850) completely abolished the binding of JAK1 to PA, whereas JAK1 with any other mutant domain showed a binding affinity similar to that of full-length JAK1 (Fig 4E and 4F), indicating that the kinase-like domain of JAK1 is the interacting domain for binding to PA.

**Fig 5:**
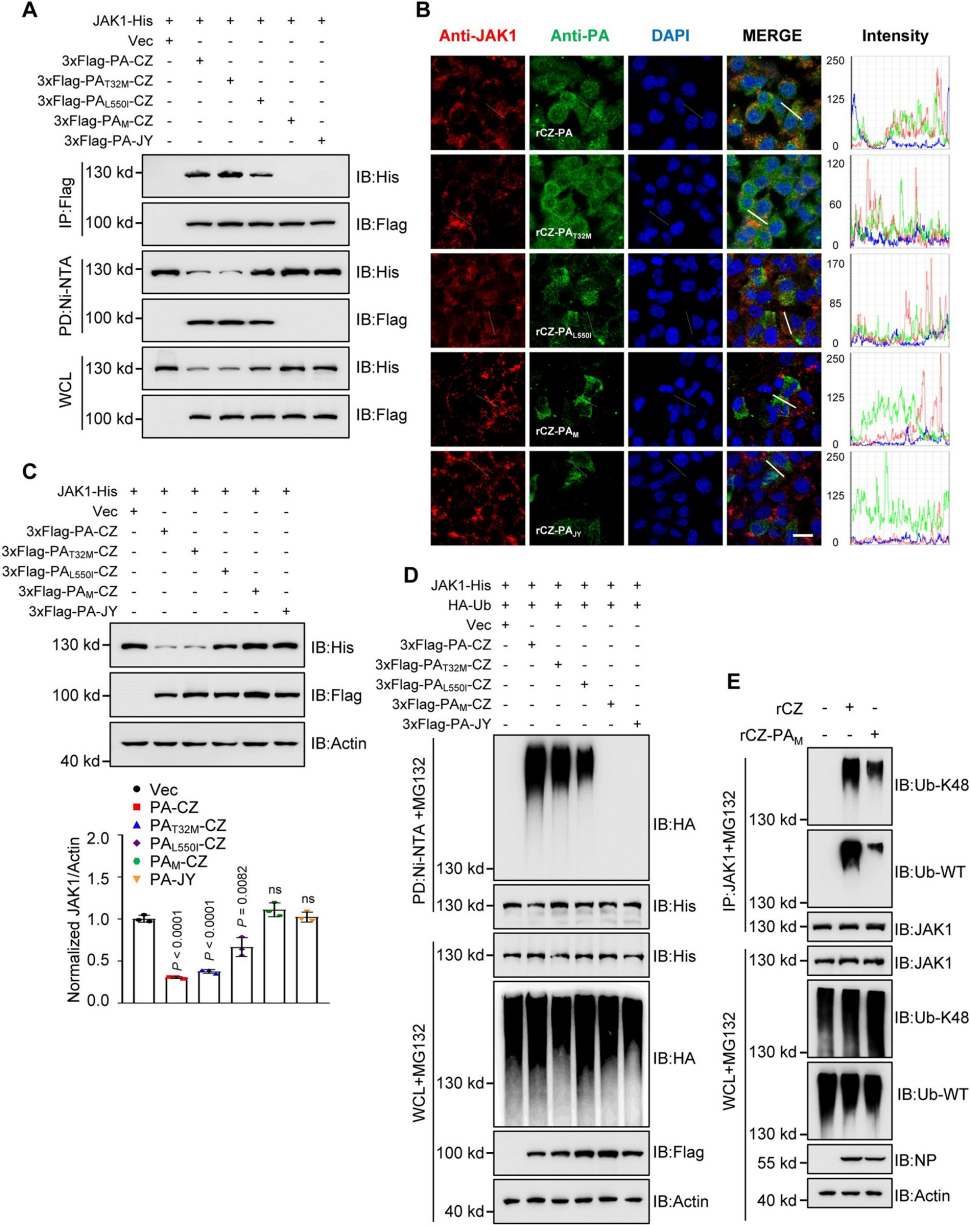
The two amino acids in PA protein mediate the interaction with and degradation JAK1. **(A)** Ni-NTA pull-down and Co-IP analysis of the interaction of JAK1 with PA and its mutants in HEK293T cells. **(B)** Co-localization of endogenous JAK1 (red) and PA (green) in rAIVs infected A549 cells. Nuclei were stained with DAPI (blue). Scale bars, 10 μm. Intensities of fluorescence at indicated locations were scanned using LAS X Software. **(C)** Immunoblots of HEK293T cells transfected with JAK1 and different PA plasmids (upper). The intensities of the bands on the immunoblots from three independent experiments were quantified and normalized with actin (lower). Statistical significance was determined using the unpaired two-tailed Student’s *t*-test. ^ns^ *P* >0.05. **(D)** Ni-NTA pull-down analysis of the ubiquitination of JAK1 in HEK293T cells transfected with PA or its mutant plasmids and treated with MG132. **(E)** Co-IP analysis of the ubiquitination of JAK1 in A549 cells infected with rAIVs at an MOI of 0.01. K at indicated residue, and K at other residues were simultaneously mutated to arginines. WCL, whole-cell lysates. Data are representative of three independent experiments.

To investigate whether the degradation of JAK1 by PA protein can be applied to various AIV strains, HEK293T cells were transfected with JAK1-His and PA from different strains. The PA proteins from selected H5, H7, and H9 subtype AIVs displayed JAK1 degradation except for that from an H5N8 strain A/duck/Eastern China/JY/2014 (JY) (S4A and S4B Fig). It is noteworthy that CZ and JY viruses differ by only two amino acids in their PA (S4C Fig). PA residues 32T and 550 L are highly conserved in human and avian IAVs (S4C and S4D Fig). To investigate the effect of 32T and 550L of PA on JAK1 interaction, PA-CZ plasmids containing T32M, L550I, and T32M/L550I (double mutations, M) mutations were constructed. IP assay revealed that the JAK1 protein bound well with PA-CZ, but not with PA_M_-CZ or PA-JY (Fig 5A). To examine the co-localization of AIV PA and JAK1 using fluorescence microscopy, we generated four recombinant CZ viruses (rCZ-PA_T32M_, rCZ-PA_L550I_, rCZ-PA_M (T32M and L550I)_ and rCZ-PA_JY_, respectively). JAK1 co-localized with PA in rCZ-infected cells (Fig 5B), but this co-localization was not observed with double substitutions (PA-T32M/L550I) in rCZ-PA_M_ or rCZ-PA_JY_ infected cells (Fig 5B). Notably, PA-CZ and PA_T32M_-CZ, but not PA_M_-CZ or PA_JY_-CZ, induced the degradation of JAK1, whereas PA_L550I_-CZ induced partial degradation of JAK1 (Fig 5C), indicating that the synergistic effect of PA 32T and 550L contributes to JAK1 degradation. Further experiments showed that PA-CZ, but not PA_M_-CZ, promoted JAK1 ubiquitination (Fig 5D). Similarly, compared to rCZ-WT, rCZ-PA_M_ infection reduced K48-linked endogenous ubiquitination of JAK1 (Fig 5E). These data suggest that the synergistic effect of PA residues 32T and 550L is critical for the ubiquitination degradation of mammalian JAK1.

The amino acid sequences of JAK1 from human and mouse are conserved, with a similarity of 94.72%, while the similarity between human and chicken JAK1 is only 81.09%. However, the Lysine 249 residue is conserved among human, mouse, and chicken (S5A Fig). Thereby, we tested whether PA degrades chicken JAK1 (chJAK1). The chJAK1 protein was significantly decreased by co-transfection of PA-CZ or PA-JY (S5B Fig), whereas neither PA-CZ nor PA-JY inhibits Rluc levels and chJAK1 transcription (S5C and S5D Fig). Moreover, both PA-CZ and PA-JY interacted with and promoted the polyubiquitination of chJAK1 (S5E and S5F Fig). These data indicate that, in contrast to degrading mammalian JAK1, the AIV PA protein with residues 32M/550I or 32T/550L can degrade chJAK1.

### The residues 32T and 550L in the PA protein play a key role in IFN-mediated signaling pathway

**Fig 6:**
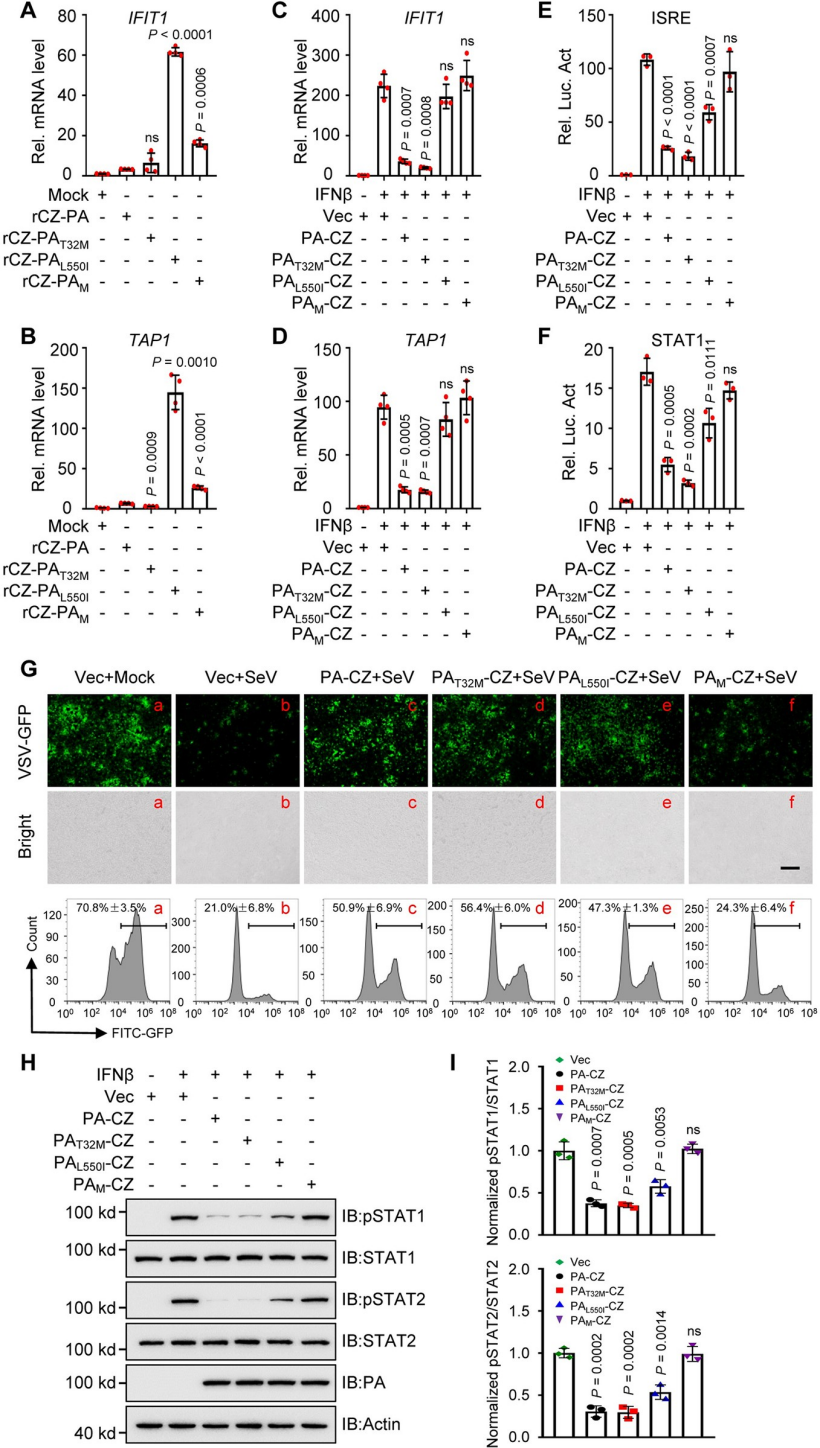
AIV PA protein inhibits IFN-mediated signaling. **(A, B)** qPCR analysis of *IFIT1* **(A)** and *TAP1* **(B)** mRNA in A549 cells infected with rAIVs (n = 4). **(C, D)** qPCR analysis of *IFIT1* **(C)** and *TAP1* **(D)** mRNA in A549 cells transfected with PA or its mutant plasmids and treated with IFNβ (n = 4). **(E, F)** Luciferase activity in HEK293T cells transfected with ISRE **(E)** or STAT1 **(F)** promoter-luciferase reporter, Renilla luciferase plasmid, and PA or its mutant plasmids and treated with IFNβ (n = 3). **(G)** HEK293T cells were transfected with PA or its mutant plasmid and infected with SeV. The supernatants were inactivated through UV and collected to treat fresh HEK293T cells for 24 h, followed by infection with VSV-GFP. The cells were observed using a microscope and then assessed using flow cytometry. Scale bars, 200 μm. **(H)** Immunoblot analysis of phosphorylated and total STAT1 or STAT2 in HEK293T cells transfected with PA or its mutant plasmid and treated with IFNβ. **(I)** Densitometry analysis of phosphorylated STAT/total STAT ratio on the immunoblots (H). Data are presented as the mean ± SD and are representative of three independent experiments. Statistical significance in **A**–**F** and **I** were determined by unpaired two-tailed Student’s *t*-test.

Next, we analyzed the levels of ISGs mRNA in A549 cells infected with the variants or WT virus. The levels of *IFIT1* (Fig 6A) and *TAP1* (Fig 6B) mRNA in A549 cells infected with the mutant viruses were higher than those in cells infected with rCZ, except for rCZ-PA_T32M_. We then explored the ability of PA to regulate the expression of ISGs. Quantitative polymerase chain reaction (qPCR) analysis further indicated that overexpression of PA-CZ and PA_T32M_-CZ, but not PA_L550I_-CZ or PA_M_-CZ, significantly inhibited IFNβ-triggered transcription of *IFIT1* and *TAP1* in human A549 cells (Fig 6C and 6D). Following IFNβ treatment, the relative activities of the ISRE and STAT1 promoters were markedly suppressed by the expression of PA-CZ and PA_T32M_-CZ but not by PA_L550I_-CZ and PA_M_-CZ (Fig 6E and 6F).

As the PA protein inhibited the transcription of ISGs, we then investigated whether the two residues in the PA protein play a role in the cellular antiviral response. As shown in Fig 6G, the ultraviolet (UV)-inactivated supernatants of PA-CZ, PA_T32M_-CZ_,_ or PA_L550I_-CZ, but not PA_M_-CZ-transfected HEK293T cells which were infected with SeV could not inhibit the replication of GFP-expressing vesicular stomatitis virus (VSV-GFP) by monitoring the GFP expression, suggesting that PA (32T/550L) protein dampened the secretion of antiviral factors induced by SeV (Fig 6G). Moreover, PA-CZ, PA_T32M_-CZ or PA_L550I_-CZ, but not PA_M_-CZ, inhibited IFNβ-induced pSTAT1 and pSTAT2 expression (Fig 6H and 6I). Collectively, these data suggest that the PA (32T/550L) protein is a negative regulator of IFN-triggered antiviral response.

### The residues 32T and 550L in the PA protein are critical for AIV replication in vitro

**Fig 7:**
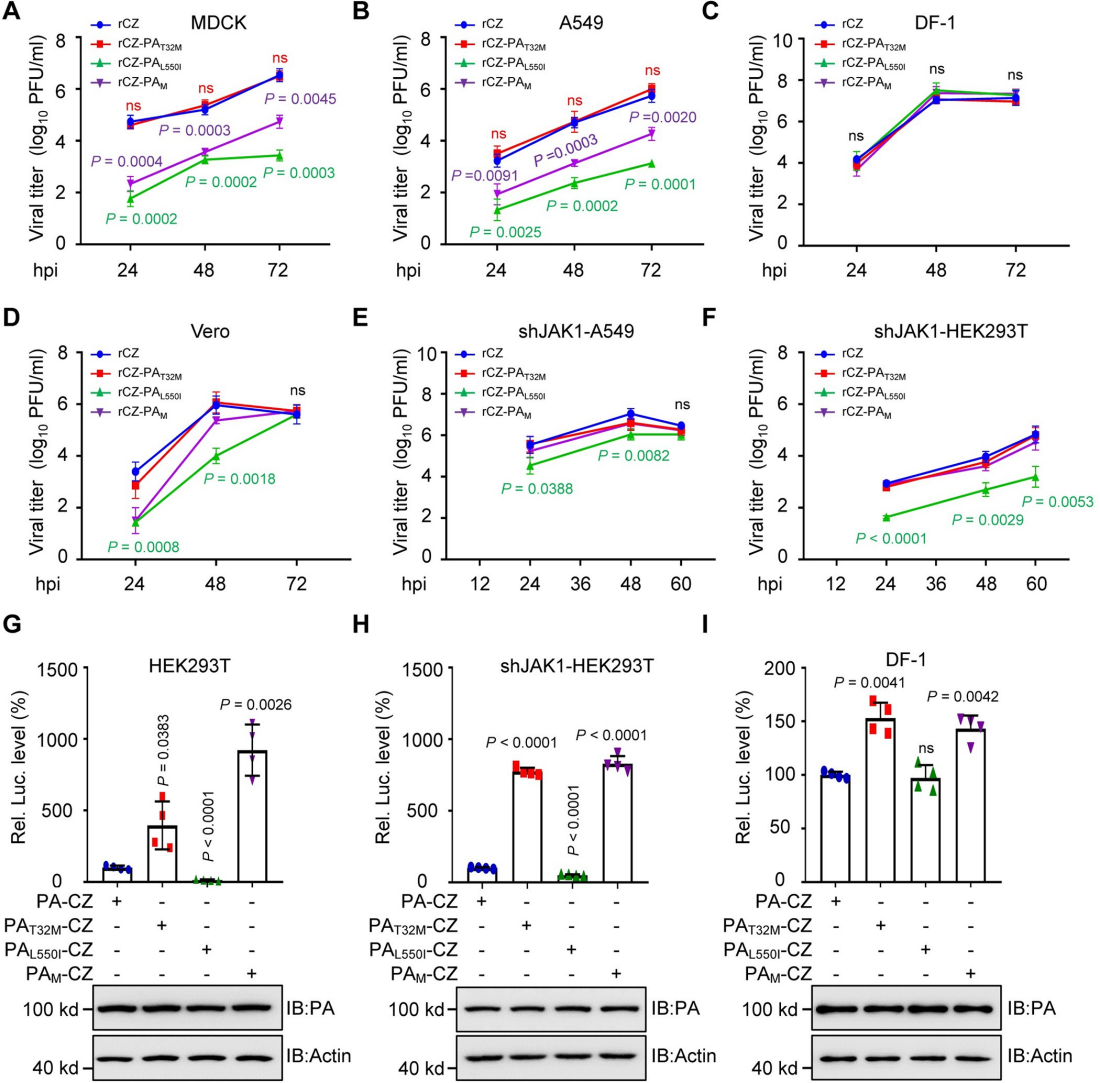
The two amino acids in PA protein are critical for replication of AIV in mammalian cells. **(A–F)** Growth curve of viruses in MDCK **(A)**, A549 **(B)**, DF-1 **(C),** Vero **(D),** shJAK1 A549 **(E)**, or shJAK1 HEK293T cells **(F)** infected with rAIVs at an MOI = 0.01 (n = 3). hpi, h post-infection. **(G**–**I)** Comparison of RNP polymerase activity for CZ RNP containing PA or its mutants in HEK293T **(G)**, shJAK1 HEK293T **(H)**, or DF-1 cells **(I)** at 24 h post-transfection (n = 4). PA protein levels estimated using immunoblotting for the minigenome assay are shown at the bottom. Data are presented as the mean ± SD. Statistical significance was determined using the unpaired two-tailed Student’s *t*-test. ^ns^ *P* >0.05.

We then examined the replication kinetics of the mutant viruses in mammalian-origin MDCK and A549 cells, and avian-origin DF-1 cells. PA mutations led to a reduction in growth titers 24–72 hours post-infection (hpi) for the rCZ-PA_L550I_ and rCZ-PA_M_ viruses, respectively, compared with the corresponding rCZ in MDCK and A549 cells (Fig 7A and 7B). All of these viruses grew similarly and efficiently in DF-1 cells and reached the maximum titers of approximately 10^7.5^ plaque-forming units (PFU)/mL at 72 hpi (Fig 7C). We further examined the replication kinetics of the mutant viruses in Vero cells, which are type I IFN-deficient [44]. The rCZ-PA virus exhibited growth kinetics similar to rCZ-PA_T32M_, and displayed a higher replication rate at 48 hpi, with viral titer reaching approximately 10^6.0^ PFU/mL. The double mutant viruses displayed slightly lower replication, and rCZ-PA_L550I_ displayed the lowest replication rate at 48 hpi. Interestingly, all mutated viruses reached titers as high as those of the WT virus at 72 hpi (Fig 7D). Further, we found that there was no significant difference in viral titers among rCZ, rCZ-PA_T32M_, and rCZ-PA_M_, and the viral titers of rCZ-PA_L550I_ were lower than that of other recombinant viruses in shJAK1 cells at 24 and 48 hpi (Fig 7E and 7F).

The PA protein is a component of the vRNP complex in AIV, which is responsible for the transcription and replication of the viral genome. Therefore, we examined the effects of the two amino acids in PA on polymerase activity in both human and avian cells. Polymerase activity was normalized against PA-CZ (set as 100%). In HEK293T cells, the polymerase activity of the single mutation PA-T32M was increased by approximately 4 fold, whereas a significant reduction in polymerase activity was observed for PA-L550I. However, the double mutation T32M/L550I in the PA protein recovered the polymerase activity, with a 9-fold increase (Fig 7G). The viral polymerase activity of PA with different mutations in shJAK1 cells was similar to that in parental cells (Fig 7G and 7H). In contrast, there was no significant change in polymerase activity found in the mutants substituted at residue 550 in DF-1 cells (Fig 7I). Only a slight increase in the viral polymerase activity was observed for PA T32M (150%) and PA T32M/L550I (150%) (Fig 7I). Further, we examined the levels of viral NP mRNA, vRNA, and cRNA in HEK293T cells or DF-1 cells infected with the recombinant viruses at 24 hpi. The results showed that there is a higher level of viral mRNA, vRNA, or cRNA in the rCZ-PA_T32M_-infected HEK293T cells than that in the rCZ-infected HEK293T cells. However, rCZ-PA_L550I_ infection has significantly lower levels of viral NP mRNA, vRNA, and cRNA than rCZ virus in HEK293T cells (S6A–S6C Fig). Interestingly, the L550I overcame the T32M increase by showing a slight restoration of the viral mRNA, vRNA, or cRNA in the rCZ-PA_M_-infected cells (S6A–S6C Fig). There was no difference in the levels of viral mRNA, vRNA, or cRNA among all the recombinant viruses in DF-1 cells (S6D–S6F Fig). These findings demonstrate that the residue 550L of PA protein is essential for polymerase activity for the transcription and replication of CZ virus in mammalian cells.

### The residues 32T and 550L in the PA protein contribute to the pathogenicity of H5 HPAIV in mice

**Fig 8:**
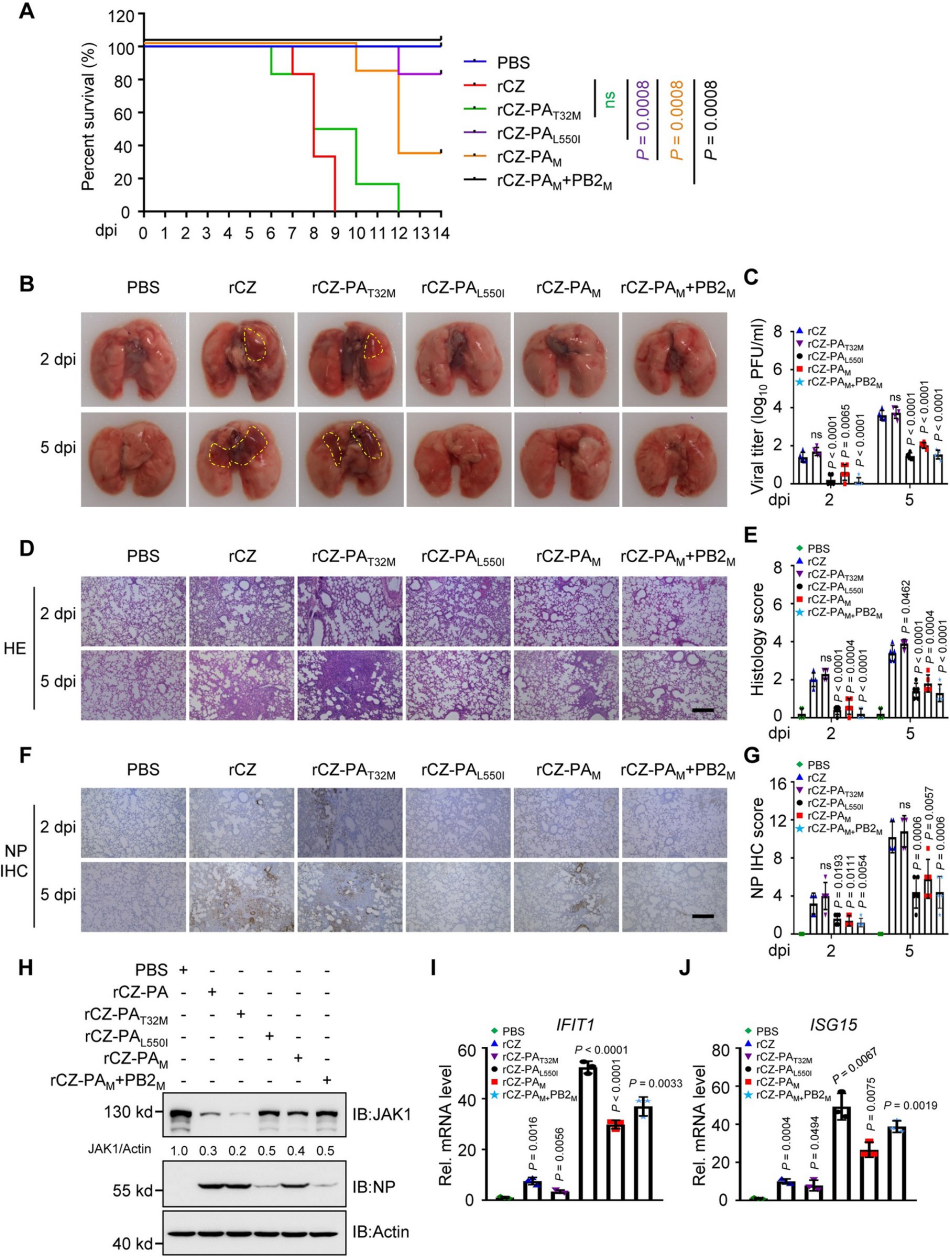
Replication and pathogenicity of rCZ and its PA mutants in mice. (**A**) The mice (n = 6 per group) were intranasally inoculated (10^3^ EID_50_ per mouse) with rAIVs and monitored for 14 d for survival. The defined humane endpoint for the mice was 20% body weight loss. **(B)** Gloss lesions of lungs from the infected mice on 2 and 5 dpi. Severe pneumonia with diffuse consolidation was outlined in yellow. The images are from representative one of five mice. **(C)** Viral titers in the lungs (n = 5 per group) were determined on 2 and 5 dpi using the plaque assay. **(D to G)** H&E staining **(D)** and scoring **(E)**, IHC staining **(F)** and scoring **(G)**. Scale bar, 200 μm. The images are from representative one of five mice and the scores were calculated from five mice. **(H)** Immunoblots of lung tissue from the infected mice. **(I, J)** qPCR analysis of *IFIT1* **(I)** and *ISG15* **(J)** mRNA in lung from the infected mice on 2 dpi (n = 3). dpi, days post-infection. Data are presented as the mean ± SD. Statistical significance was determined using the unpaired two-tailed Student’s *t*-test in **C, E, G, I,** and **J**, or *log-rank* test in **A**. ^ns^ *P* >0.05.

We further investigated whether these two mutations in PA contribute to viral pathogenicity. Mice infected with rCZ-PA_L550I_ or rCZ-PA_M_ exhibited lower morbidity than those infected with rCZ (Fig 8A). Notably, severe pneumonia was observed through gross and histopathological lesions in the lung tissues of mice inoculated with rCZ at 2 and 5 days post-infection (dpi), characterized by consolidation, hemorrhages, and edema (Fig 8B). In contrast, minimal lung consolidation was observed in mice infected with rCZ-PA_L550I_ or rCZ-PA_M_ at 2 dpi (Fig 8B). Lung virus titers were determined at 2 and 5 dpi, and were lower in rCZ-PA_L550I_ and rCZ-PA_M_ inoculated mice than in rCZ-inoculated mice, with a 1.6–2.1 log_10_ PFU/mL decrease at 5 dpi (Fig 8C). The rCZ-PA_L550I_ viral titer in the lungs was significantly lower than that in rCZ-WT-infected mice (Fig 8C). The effects of mutations on the pathogenicity of the rCZ virus in mice were further demonstrated by hematoxylin and eosin (H&E) staining of lung tissues collected at 2 and 5 dpi. Mild or moderate broncho-pneumonitis was observed in the lungs of mice infected with rCZ-PA_L550I_ or rCZ-PA_M_ (Fig 8D and 8E). The rCZ-WT-infected lungs exhibited the most severe broncho-pneumonitis and localized interstitial pneumonia (Fig 8D and 8E). In addition, viral protein NP staining was more intense in the lung sections of mice infected with rCZ than in those infected with rCZ-PA_L550I_ or rCZ-PA_M_ virus (Fig 8F and 8G). We found that the replication and pathogenicity of rCZ-PA_T32M_ were similar to those of rCZ virus.

We previously found that amino acid residues 283M and 526R in PB2 are responsible for enhancing the virulence of CZ in mice [26,36]. We hypothesized that a synergistic effect may be observed if certain PA and PB2 mutations were introduced simultaneously in the CZ background. Thus, rCZ-(PA_M_+PB2_M_), a panel of two double mutants, was generated, with PA T32M and PA L550I coupled with PB2 M283I and PB2 K526R. rCZ-(PA_M_+PB2_M_) replicated with virus titers significantly lower than those of rCZ-WT and other mutant viruses at 2 and 5 dpi (Fig 8C). The pathogenicity of rCZ with mutations in both PA and PB2 segments (PA_M_+PB2_M_) was attenuated compared with the rCZ-PA_M_ virus (Fig 8A–8G).

Moreover, the expression of JAK1 was lower in the lungs of mice infected with rCZ or rCZ-PA_T32M_ than in the lungs of mice infected with rCZ-PA_L550I_, rCZ-PA_M_ or rCZ-(PA_M_+PB2_M_) (Fig 8H). We also analyzed the levels of ISGs mRNA in the lungs of mice infected with the variants and WT. At 2 dpi, the levels of *IFIT1* (Fig 8I) and *ISG15* (Fig 8J) mRNA in mice infected with rCZ-PA_L550I_, rCZ-PA_M_ or rCZ-(PA_M_+PB2_M_) were higher than those in mice infected with rCZ or rCZ-PA_T32M_.

Collectively, these results demonstrate that the residues 32T and 550L in the PA protein of H5 HPAIV contribute to pathogenicity in mice.

## Discussion

AIV can overcome interspecies barriers under suitable conditions and infect mammals, including humans, resulting in mild to severe respiratory tract infections. This study identified two residues in the PA protein of H5 subtype HPAIV that contribute to pathogenicity in mammals, which confer inhibited IFN-mediated immune response by degrading JAK1 at the lysine 249 residue and maintaining optimum polymerase activity in mammalian cells.

Type I IFN-mediated innate immune response serves as the first line of defense against invading pathogens, including IAV. Host proteins can inhibit IAV replication by interacting with viral proteins and positively regulating type I IFN response [19,45]. The influenza virus NS1 protein is known to be a multifunctional protein that is important for the regulation of viral replication and inhibition of the host antiviral response [46]. NS1-deficient virus-infected cells significantly increased the production of type I IFN [47], suggesting that the importance of other viral proteins in the inhibition of type I IFN cannot be ignored. Type I IFN is secreted and activates the JAK/STAT signaling pathway, thereby inducing the expression of ISGs that can repress viral replication [16]. Numerous viruses, including Zika virus (ZIKV) [48] and SARS-CoV-2 [49], target JAK1 for degradation, suppress JAK/STAT signaling, and impair the IFN-mediated antiviral response. Recently, we showed that the IAV PB2 protein inhibits the type I IFN-inducing pathway by binding to JAK1 [26]. The present study further demonstrated that the AIV PA protein, independent of the PB2 protein, impairs the JAK/STAT signaling pathway by targeting and degrading JAK1 in a proteasome-dependent manner, thereby reducing the phosphorylation levels of STAT1/STAT2, which creates a cellular environment favorable for the replication and propagation of influenza virus. Furthermore, PA residues 32T and 550L are critical for degrading mammalian JAK1. As expected, PA (32T/550L) markedly inhibited the mRNA abundance of ISGs in response to IFNβ treatment. However, both PA-CZ (32T/550L) and PA-JY (32M/550I) efficiently interacted with chJAK1 for ubiquitination and degradation. PA 32T and PA 550L are also highly conserved in IAVs of avian and human origin, suggesting an important role for these amino acids in the polymerase context. It is conceivable that sequence variations in the PA proteins of different viral strains influence the affinity for JAK1 binding, which may correlate with viral pathogenesis.

JAKs contain a conserved domain organization consisting of N-terminal FERM and putative SH2 domains, followed by kinase-like and kinase domains [50]. The activity of the C-terminal kinase domain is essential for signaling, whereas the kinase-like domain has been shown to regulate cytokine-induced activation [51]. The FERM and SH2 domains mediate the association with cytokine receptors [52]. The kinase-like and kinase domains of JAK1 interact with PB2, and lysine residues 859 and 860 in the kinase domain of JAK1 are critical for efficient PB2-mediated degradation [26]. In this study, detailed biochemical analysis further revealed that the kinase-like domain is associated with PA. The lysine 249 residue in the FERM domain of mammalian JAK1 is critical for efficient PA-mediated ubiquitination degradation. The ubiquitination site at lysine 249 of JAK1 was also identified by MS during SARS-CoV-2 infection [53]. Interestingly, ubiquitin ligase STUB1 mediates ubiquitination at lysine 249 and promotes JAK1 degradation [54]. Although mutation of K249R in JAK1 led to its degradation resistance, these mutations also weakened the JAK1-mediated antiviral immune response (Fig 3). These data suggest that lysine 249 is essential for both JAK1 ubiquitination and JAK1-mediated ISGs antiviral activity. Even though residue 249 of chJAK1 is lysine as well, PA containing 32M/550I residues mediates the degradation of chJAK1, but not mammalian JAK1, which suggests that there may be host factors involved in the degradation process of JAK1 by PA protein. Future studies will determine whether STUB1 or other host factors are exploited by PA protein to promote JAK1 ubiquitination degradation at the lysine 249, therefore providing new targets for IAV treatment.

By characterizing the effect of L550I of PA protein on the replication of AIV, we found that the growth kinetics of viruses carrying L550I were severely compromised in MDCK and A549 cells (Fig 7A and 7B), but not in avian-origin DF-1 cells (Fig 7C). The ribonucleoprotein (RNP) complex polymerase activity of IAV is critical for the transcription and replication of the viral genome. PA L550I reduced viral polymerase activity in mammalian-origin HEK293T cells but not in avian-origin DF-1 cells, confirming that the L550I in PA protein can reduce AIV polymerase activity in mammalian cells (Fig 7G–7I), which accounts for the virus containing the L550I mutation, grew slower and to a lower titer than the WT virus. However, although the T32M or T32M/L550I mutations enhanced polymerase activity more than the WT, the enhanced polymerase activity did not directly reflect the multistep growth of the mutant virus. Therefore, the viral replication is not always correlated with the polymerase activity, which is similar to a prior study in which the excessive polymerase activity did not lead to high replication and virulence of H7N7 recombinant viruses in mice [55]. Importantly, rCZ-PA and rCZ-PA_M_ replication dynamic became similar in shJAK1 cells (Fig 7E and 7F), suggesting that the regulation of PA (32T/550L) protein on viral replication is primarily by antagonizing JAK1-mediated antiviral immunity.

Mutations in the polymerase complex are critical for influenza viruses to overcome host barriers and achieve cross-species transmissions. A major determinant of species tropism is the identity of amino acid 627 in the influenza polymerase PB2 subunit [56]. In contrast, the role of PA in host adaptation has not been as well characterized as that of PB2. However, it was reported that an arginine-to-lysine substitution at position 185 of the avian influenza virus H5N1 PA protein affected the virulence and pathogenicity of the virus in mice [57]. A recent study indicated that the PA gene plays a major role in the difference in pathogenicity between two swine influenza viruses in mice [58]. Here, we identified two additional residues in the PA protein that affect virulence: 32T and 550L. The virulence of the PA 32M/550I viruses was attenuated compared to that of the 32T/550L viruses, which indicates that these PA residues are pathogenicity factors in the mammalian host (Fig 8). The PA T32M/L550I mutant virus with weak JAK1 degradation ability replicated lower titers in mouse organs and mammalian cells than that of the corresponding WT virus, which may be primarily caused by the high expression of ISGs. Consistent with the in vitro growth properties, the L550I mutant virus showed a decrease in viral replication in vivo, which was mainly due to the inhibition of polymerase activity. Furthermore, the inhibition of JAK1 degradation and high expression of ISGs in infected mice resulted in decreased pathogenicity of the L550I mutant virus in mice. Moreover, several other adaptive mutations in PB2 (K526R and M283I/K526R) can enhance the virulence of H5 or H7 AIVs in mammals [35,36]. Since the 283M and 526K in PB2 protein are critical for JAK1 degradation [26], and the PA-mediated JAK1 degradation was intensified by the presence of PB2 protein (S2G Fig), *in vivo* study indicated that the addition of the PB2 M283I/K526R mutations decreased the pathogenicity by comparing to the virus that carries the PA T32M/L550I mutation alone in mice. However, only the L550I mutation decreased morbidity compared to T32M/L550I viruses, as evidenced by lung pathology, as well as the lung viral titer after infection (Fig 8). These results are in agreement with those of a previous study, where I550L in the PA protein contributes to the high virulence of PR8 influenza virus [59].

Notably, the PA gene also encodes a novel protein, PA-X, a fusion protein that integrates the N-terminal 191 amino acid leader sequence originating from PA and a C-terminal region of the ribosomal frame-shifting product that lengthens to 41 or 61 amino acids encoded by an overlapping open reading frame (ORF) (“X-ORF”) [60]. PA-X plays an important role in host shutoff, inhibiting cellular gene expression and hindering the induction of antiviral responses [60]. PA did not affect the expression of eGFP and Rluc. Furthermore, the residue 550L on the C-terminal of PA, not on the N-terminal of PA (PA-X), and the synergistic effect of PA residues 32T and 550L on the degradation of JAK1 (Fig 5) indicate that the AIV PA, independent of PA-X protein, specifically degrades JAK1 protein and contributes to viral pathogenicity in mice.

In summary, we found that the two residues in the PA protein, 32T, and 550L, are critical for mediating mammalian JAK1 degradation and maintaining polymerase activity, which contributes to the virulence of AIV in mammals. Our study provides important insights into the development of rational antiviral and vaccine strategies.

## Materials and methods

### Ethics statement

Animal experiments were conducted in accordance with the guidelines for experimental animal welfare and ethics. All animal studies followed the protocols of the Jiangsu Province Administrative Committee for Laboratory Animals (permit number: SYXK-SU-2017-0044). All experiments on influenza virus infection were conducted at a biosafety level 3 laboratory and animal facility at Yangzhou University (CNAS BL0015).

### Viruses

Two HPAI H5N8 viruses, A/goose/eastern China/CZ/2013 (CZ) and A/duck/Eastern China /JY/2014 (JY), were characterized in our previous studies [61]. Recombinant viruses were rescued by reverse genetics using eight plasmid-based reverse genetic systems as described previously [62]. Eight segments from the CZ strain were cloned into the bidirectional reverse-genetics plasmid, pHW2000. The CZ backbone was used to rescue recombinant viruses with various PA or PB2 genes containing different mutants, named rCZ-PA_T32M_, rCZ-PA_L550I_, rCZ-PA_(M, T32M/L550I),_ rCZ-PA_JY_, and rCZ-[PA_(M, T32M/L550I)_+PB2_(M, M283I/K526R)_]. The rescued viruses were confirmed using sequence analysis, and viruses bearing unwanted mutations were excluded from further analysis.

### Cells and virus infection

Human embryonic kidney (HEK) 293T cells (ATCC, CRL-11268), chicken fibroblast cell line DF-1 cells (ATCC, CRL-12203), and human lung epithelial A549 cells (ATCC, CCL-185) were cultured in Dulbecco’s modified Eagle’s medium (DMEM; HyClone, USA). Madin-Darby canine kidney (MDCK) cells (ATCC, CCL-34) cells were cultured in minimum essential medium Eagle (MEM). ShJAK1 HEK293T/A549 cells were prepared as previously described [26]. All media were supplemented with 10% fetal bovine serum (FBS), and the cells were routinely cultured at 37°C with 5% CO_2_. Cells were inoculated with AIV at the indicated multiplicity of infection (MOI) for virus infection. After adsorption for 1 h at 37°C, the cells were washed with phosphate-buffered saline (PBS) and cultured in DMEM/MEM containing 0.5 μg/mL L-1-tosylamido-2-phenylethyl chloromethyl ketone (TPCK)-treated trypsin.

### Titrations

Viral loads were determined using plaque assays in MDCK cells. The cells were washed with PBS for 1 h after inoculation and replaced with 2×DMEM mixed with an equal portion of 1% agarose containing 0.5 μg/mL TPCK-treated trypsin. After 72 h of incubation, MDCK cells were fixed with 4% formaldehyde and stained with crystal violet.

### Reagents and antibodies

Proteasome inhibitor MG132 (Beyotime Biotechnology), chloroquine (CQ; Sigma-Aldrich), lysosome inhibitor NH4Cl (Fisher Scientific), cycloheximide (CHX, Sigma-Aldrich), 4’, 6-diamidino-2-phenylindole dihydrochloride (DAPI; Sigma-Aldrich), the dual-luciferase reporter assay system (Promega and Vazyme), recombinant human IFNβ (GenScript), and anti-Flag M2 affinity agarose (Sigma-Aldrich) were purchased from the indicated manufacturers. Mouse anti-NP mAb (GTX629633, 1:1000), rabbit anti-PA pAb (GTX125932, 1:1000), rabbit anti-PB1 pAb (GTX125923, 1:1000) and rabbit anti-PB2 pAb (GTX125926, 1:1000) were purchased from GeneTex. Rabbit anti-STAT1 mAb (14995S, 1:1000), rabbit anti-pSTAT1 mAb (8826S, 1:1000), rabbit anti-STAT2 mAb (72604S, 1:1000), rabbit anti-pSTAT2 mAb (88410S, 1:1000), rabbit anti-K48-linkage specific polyubiquitin mAb (8081S, 1:500), mouse anti-HA-tagged mAb (2367S, 1:500), rabbit anti-JAK1 mAb (29261S, 1:1000), and mouse anti-JAK1 mAb (50996S, 1:1000) were purchased from Cell Signaling Technology. Mouse anti-beta actin mAb (Santa Cruz, sc47778, 1:2000), mouse anti-JAK1 mAb (Zen BIO, 200622-8B8, 1:500), rabbit anti-IFN alpha/beta receptor 1 pAb (HUABIO, ET1602-37, 1:500), rabbit anti-Ub mAb (HUABIO, ET1609-21, 1:500), mouse anti-His tag mAb (MBL, D291-3MS, 1:2000), and mouse anti-FLAG M2 mAb (Sigma-Aldrich, F1804, 1:2000) were purchased from the indicated manufacturers. Alexa Fluo 594 Goat anti-Mouse IgG (H+L) (ab150116, 1:400) and Alexa Fluor 488 Goat anti-Rabbit IgG (H+L) (ab150077, 1:400) were purchased from Abcam.

### Plasmid construction and transfection

All plasmids were constructed using standard molecular biology techniques. Viral genes were amplified from the IAV genomes using reverse transcription (RT)-PCR and cloned into 3×Flag-CMV-10 or pHW2000. The site-directed mutagenesis kit (Vazyme) was used to generate *JAK1* or *PA* gene mutations. Primer sequences used for cloning are available upon request. All constructs were validated by sequencing and transfected into cells using the Transfection Reagent (BioBEST). The total amount of DNA plasmids in each transfection was kept constant by adding a corresponding empty vector (Vec).

### RT-qPCR analysis

Total RNA was extracted using TRIzol reagent (Sigma-Aldrich) and reverse-transcribed using the SuperScript cDNA synthesis kit (Invitrogen), according to the manufacturer’s instructions. qPCR was performed using the SYBR Green kit (Thermo Scientific). The generated cDNA was subjected to qPCR in a 20 μl reaction volume using gene-specific primers. The cDNA quantities were normalized to GAPDH/Actin. The qPCR primers used in this study are listed in S1 Table, as previously described [26].

### VSV-GFP bioassay

Antiviral cytokine secretion bioassays were conducted as previously described [26,63], with slight modifications. HEK293T cells were transfected with the indicated plasmids. At 18 h post-transfection (hpt), cells were infected with SeV (MOI = 1) for an additional 24 h. The supernatants were harvested and inactivated by placing the samples under a 30-W UV lamp for 20 min. UV-inactivated supernatant was added to fresh confluent cells and incubated for 24 h. The cells were infected with VSV-GFP at an MOI of 0.1. At 24 hpi, VSV-GFP replication was visualized by monitoring the GFP expression level using fluorescence microscopy or flow cytometry.

### Confocal immunofluorescence assay

Confocal dish-adhered A549 cell monolayers were infected with AIV for 18 h. Treated cells were washed twice with phosphate-buffered saline-Tween 20 (PBST), fixed in 4% paraformaldehyde, permeabilized with 0.2% Triton X-100, air-dried, and blocked with PBS containing 5% skimmed milk. The fixed cells were then incubated with the indicated primary antibodies at 37°C for 1 h, rinsed, and incubated with the corresponding secondary antibodies. The nuclei were counterstained with DAPI. Images were captured using a Leica SP8 confocal microscope and processed using LAS X Software (V3.7.4).

### Co-IP

The co-IP assay was conducted as previously described with minor modifications [26,64]. Briefly, HEK293T cells transfected with the indicated plasmids or A549 cells infected with AIV were harvested and lysed with NP-40 lysis buffer. Lysates were incubated overnight at 4°C with the indicated antibodies followed by incubation with protein A/G agarose beads (Santa Cruz Biotechnology) for 2 h at 4°C. After four stringent washes with NP-40 buffer, immunoprecipitates were subjected to immunoblotting analysis.

### Ni-NTA pull-down assays

The Ni-NTA pull-down assays were performed as previously described [20,26]. HEK293T cells transfected with the indicated plasmids or infected with AIV were harvested, resuspended in buffer A (6 M guanidine-HCl, 0.1 M Na_2_HPO4/NaH_2_PO4, 10 mM Tris-Cl, pH 8.0, 5 mM imidazole, and 10 mM β-mercaptoethanol), and sonicated for 30 s. Cell lysates were incubated with 40 μL of pre-equilibrated Ni-NTA beads (QIAGEN) overnight at 4°C. The beads were washed four times sequentially with buffers A and B (8 M urea, 0.1 M Na_2_HPO4/NaH_2_PO4, 10 mM Tris-HCl, pH 8.0, and 10 mM β-mercaptoethanol), and C (same as B, except pH 6.3). The precipitates were boiled in an SDS sample buffer containing 200 mM imidazole and subjected to immunoblotting analysis.

### Luciferase assay

HEK293T cells were transfected with a mixture of luciferase reporter (IFNβ-Luc, ISRE-Luc, or STAT1-Luc) and pRL-TK (Renilla luciferase plasmid) together with the indicated plasmids. At 30 hpt, cells were treated with SeV or IFNβ for an additional 12 h. The cells were lysed with a passive lysis buffer, and luciferase activity in the lysates was determined using the dual-luciferase reporter assay system. The Renilla luciferase construct pRL-TK was transfected as an internal control.

A minigenome assay based on a dual-luciferase system was performed as previously described [36]. Briefly, HEK293T cells were transfected with PB2, PB1, NP, and PA (originating from the influenza virus CZ strain) or its mutants (PA_T32M_, PA_L550I_, and PA_T32M/L550I_), the luciferase reporter plasmid p-Luci, which expresses reporter vRNA encoding the firefly luciferase gene, and pRL-TK-RLuc, which expresses Renilla luciferase as an internal control. For the luciferase reporter assays in DF-1 cells, a luciferase reporter containing a chicken RNA polymerase I (Pol I)-driven promoter was constructed. Luciferase activity was measured using the dual-luciferase reporter assay system. Polymerase activity was calculated by normalizing the firefly luciferase activity to the Renilla luciferase activity. The polymerase activity of the WT was set to 100%.

### Mouse study

Five-week-old female BALB/c mice were purchased from the Yangzhou Experimental Animal Center, Yangzhou, China, and housed in individually ventilated cages. The mice were maintained on a 12/12-h light/dark cycle at 25°C and 40–60% relative humidity. The mice were 6 weeks old at the time of infection. To evaluate the pathogenicity of the viruses, groups of six mice were inoculated intranasally with 50 μL of the indicated dose of each influenza virus diluted in PBS.

Mice were intranasally inoculated with 10^3.0^ EID_50_ of each indicated virus. Animals were observed daily for mortality up to 14 dpi. At 2 and 5 dpi, five mice from each group infected with each influenza virus or mock-infected with PBS were euthanized, and lung tissues were collected from each mouse for virus titration, immunoblotting, histochemical staining, and RT-qPCR.

### Lung tissue histology and immunohistochemistry (IHC) assay

The lungs from control or virus-infected mice were dissected, fixed in 10% formalin, embedded in paraffin, sectioned, stained with H&E solution, and visualized using a light microscope to assess histological changes. The lung histological score was assessed by a pathologist in a blinded manner following a standardized scoring system, as previously described [26,65]. Briefly, lung microscopic lesions were blindly evaluated from 0 to 4 in three random areas to account for the distribution and severity of interstitial pneumonia. For IHC assays, the tissue sections were stained with an anti-influenza virus NP monoclonal antibody. According to the percentage of positive cells, the proportion score of NP expression was classified as follows: 0, 0%; 1, ≤10%; 2, 11–50%; 3, 51–80%; and 4, ≥81%. Staining intensity was evaluated as negative (0), mild (1), moderate (2), and strong (3). The total score was calculated by multiplying the intensity score with the proportion score [26,66].

### Statistical analysis

GraphPad Prism software V8.0.1. was used for statistical analyses. Data are presented as the mean ± standard deviation (SD), unless otherwise indicated. The Student’s *t*-test was used for two-group comparisons. Statistical significance was set at *P* < 0.05 (*) and *P* < 0.01 (**). The Kaplan-Meier method was used for animal survival analysis to generate graphs, and the survival curves were analyzed with log-rank analysis.

## Supporting information

S1 Fig Screening of IAV proteins for their effect on the expression of JAK1. (**A–J**) HEK293T cells were transfected with plasmids encoding viral proteins (PB2, PB1, PA, HA, NP, NA, M1, NS1, M2, and NS2) and JAK1 for 36 h before IB analysis. The intensities of the indicated protein bands were determined by using image J, were normalized to Actin, and are shown as the fold-change of JAK1/Actin. Data are representative of two independent experiments.
(TIF)

S2 Fig AIV PA protein specifically degrades JAK1. **(A–C)** Immunoblots of HEK293T cells were transfected with indicated plasmids, and the amount of each plasmid is indicated above the lanes (in microgram) (upper). The intensities of the bands on the immunoblots from three independent experiments were quantified and normalized with actin (lower). **(D, E)** HEK293T cells were transfected with plasmids encoding PA from CZ, together with GFP **(D)** or Rluc **(E)** expression vectors. Cells expressing GFP were observed using an inverted fluorescence microscope under identical exposure conditions and then assessed by flow cytometry. Cells expressing Rluc were performed by Luciferase assays. **(F)** qPCR analysis of *JAK1* mRNA level in A549 cells transfected with different concentrations of PA plasmids (from 0.5, 1 to 1.5 μg) (n = 4). **(G)** Immunoblots of HEK293T cells were transfected with indicated plasmids, and the amount of each plasmid was indicated above the lanes (in microgram) (upper). The intensities of the bands on the immunoblots from three independent experiments were quantified and normalized with actin (lower). **(H)** Immunoblots of HEK293T cells infected with CZ virus at an MOI of 0.1 (left). The intensities of the bands on the immunoblots from three independent experiments were quantified and normalized with actin (right). hpi, h post-infection. **(I)** Immunoblots of HEK293T cells treated with different concentrations of IFNβ (from 10, 100 to 1000U/ml) (left). The intensities of the bands on the immunoblots from three independent experiments were quantified and normalized with actin (right). Data are presented as the mean ± SD. Statistical significance was determined by unpaired two-tailed Student’s *t*-test in **A**–**C, E–I**. ^ns^ *P* >0.05.
(TIF)

S3 Fig AIV PA protein increases the K48-linked ubiquitination of JAK1. Ni-NTA pull-down analysis of the ubiquitination of JAK1 in HEK293T cells transfected with JAK1, HA-Ub or its mutants [K at indicated residue, and K at other residues were simultaneously mutated to arginines], and PA plasmids and treated with MG132. Data are representative of two independent experiments.
(TIF)

S4 Fig Two amino acids in PA protein are critical for the degradation of JAK1. **(A)** Immunoblots of HEK293T cells transfected with different IAV PA plasmids. **(B)**. Quantification of protein expression on immunoblots (A) and normalized with actin (n  =  3). Data are presented as means ± SD and statistical significance was determined by unpaired two-tailed Student’s *t*-test. **(C)** The amino acid differences between the PA protein from different IAVs. **(D)** Frequencies of different residues at PA 32 and 550 positions in avian and human viruses. All sequences were obtained from GISAID database.
(TIF)

S5 Fig AIV PA protein mediates ubiquitination degradation of chicken JAK1 (chJAK1). **(A)** Amino acid alignment of JAK1 from the different species. The black box highlights amino acid 249 corresponding to human JAK1. **(B)** Immunoblots of HEK293T cells transfected with PA and chJAK1 plasmids (upper). Densitometry analysis of the ratio of JAK1/Actin on immunoblots from three independent experiments (lower). **(C)** Luciferase assays in DF-1 cells transfected with Rluc plasmid, and PA-CZ or PA-JY plasmid (n = 3). **(D)** qPCR analysis of *chJAK1* mRNA level in DF-1 cells transfected with PA plasmids from CZ and JY. (n = 3). **(E)** Co-ip analysis of the interaction of PA with chJAK1 in HEK293T cells. **(F)** Ni-NTA pull-down analysis of the ubiquitination of chJAK1 in HEK293T cells transfected with chJAK1, HA-Ub, and PA plasmids. WCL, whole-cell lysates. Data are presented as means ± SD and statistical significance was determined by unpaired two-tailed Student’s *t*-test in **B**–**D**. ^ns^ *P* >0.05. Data are representative of three independent experiments.
(TIF)

S6 Fig Quantitative analysis of *mRNA, vRNA*, and *cRNA* levels in rAIVs-infected cells. **(A–C)** HEK293T cells or **(D–F)** DF-1 cells were infected with rAIVs. Levels of NP genes were estimated by quantitative RT-PCR (n = 3). Data are presented as means ± SD and statistical significance was determined by unpaired two-tailed Student’s *t*-test. ^ns^ *P* >0.05.
(TIF)

S1 Table The qPCR primers sequence in this study. (TIF)
