# Supplementary figures and images for: The synergistic effect of residues 32T and 550L in the PA protein of H5 subtype avian influenza virus contributes to viral pathogenicity in mice
Source: PLoS Pathog. 2023 Jul 3;19(7):e1011489. doi: 10.1371/journal.ppat.1011489 (PMC10348578; doi:10.1371/journal.ppat.1011489)

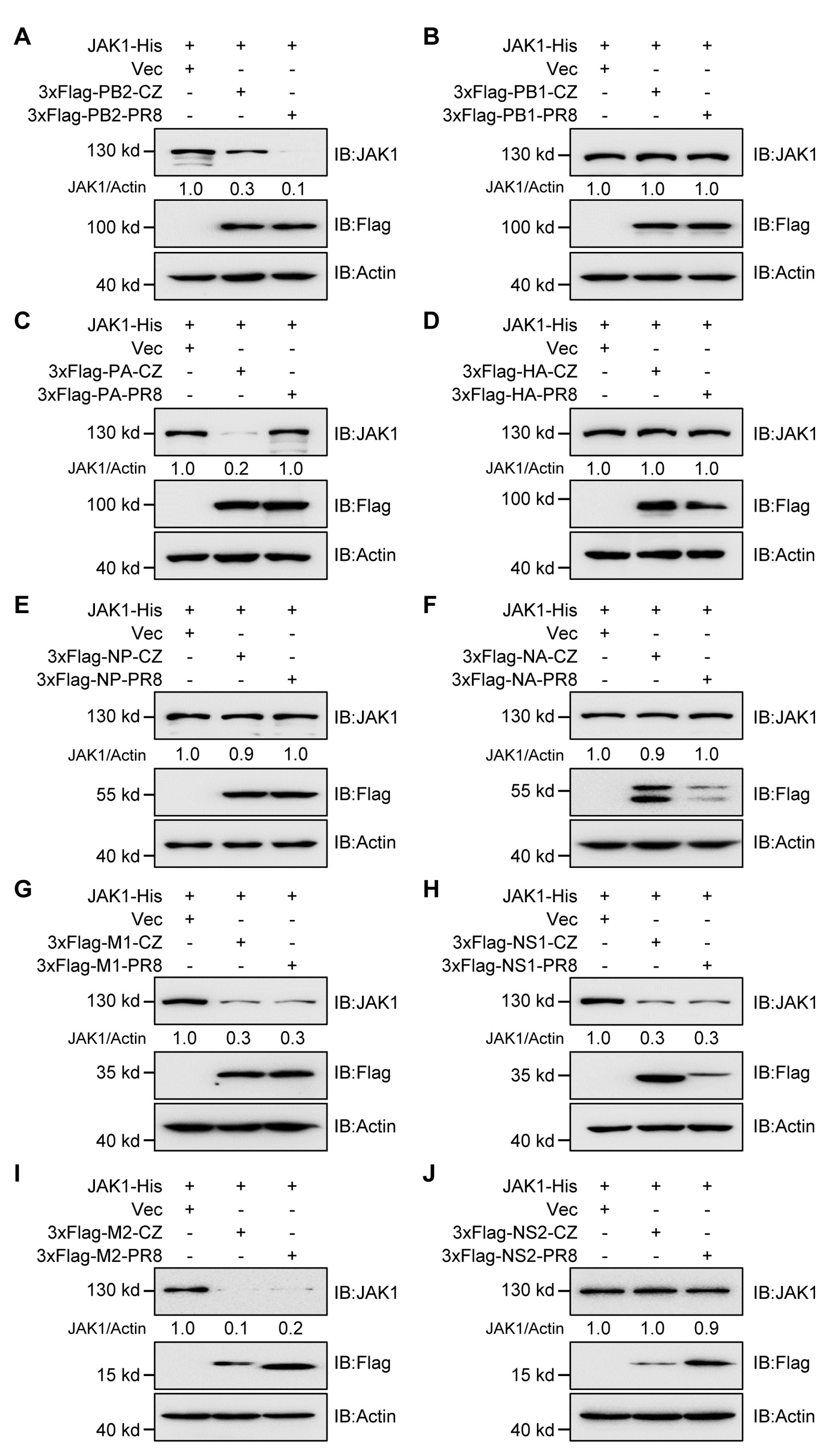

Supplement: S1 Fig — (A–J) HEK293T cells were transfected with plasmids encoding viral proteins (PB2, PB1, PA, HA, NP, NA, M1, NS1, M2, and NS2) and JAK1 for 36 h before IB analysis. The intensities of the indicated protein bands were determined by using image J, were normalized to Actin, and are shown as the fold-change of JAK1/Actin. Data are representative of two independent experiments. (TIF) [file ppat.1011489.s001.tif]

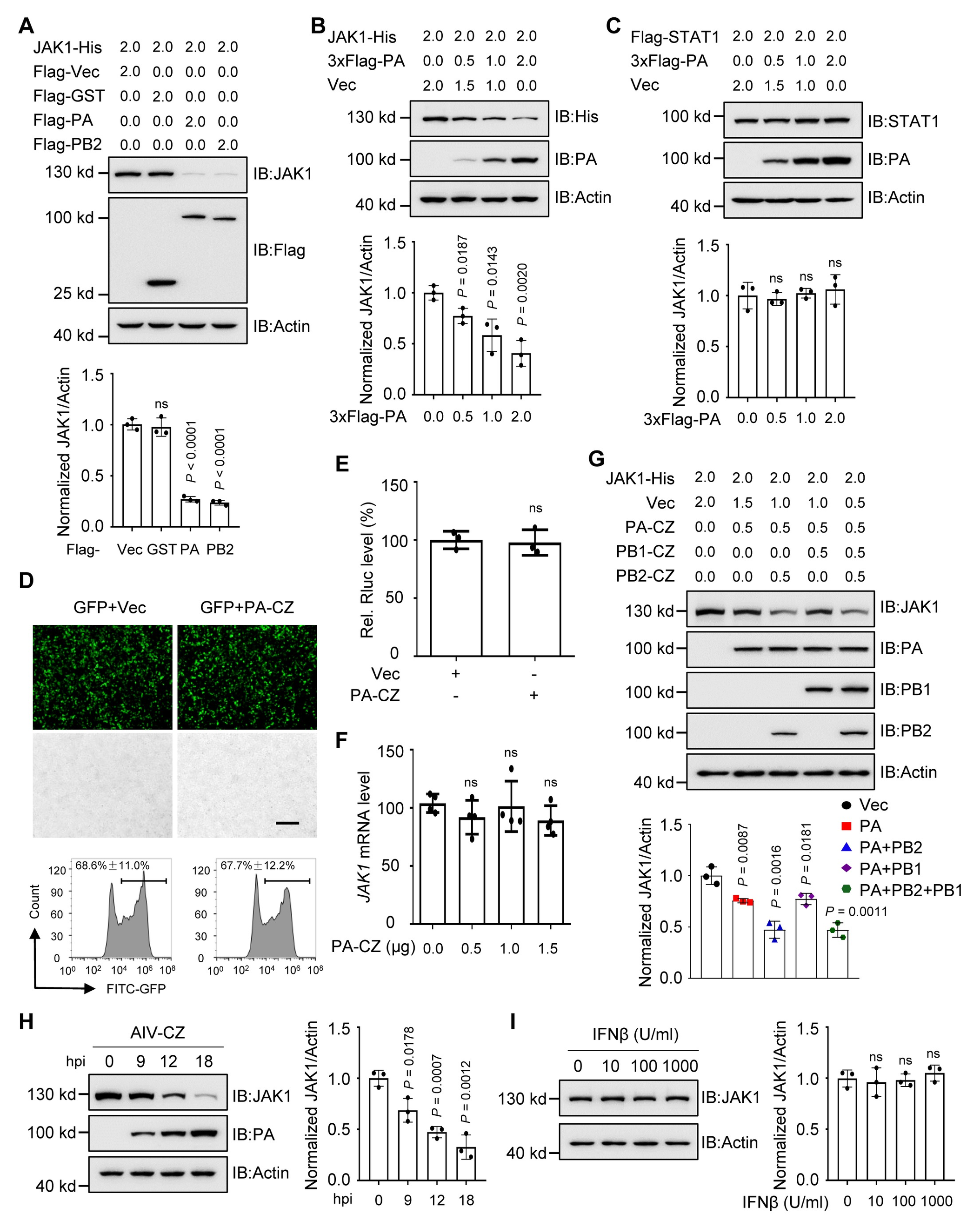

Supplement: S2 Fig — (A–C) Immunoblots of HEK293T cells were transfected with indicated plasmids, and the amount of each plasmid is indicated above the lanes (in microgram) (upper). The intensities of the bands on the immunoblots from three independent experiments were quantified and normalized with actin (lower). (D, E) HEK293T cells were transfected with plasmids encoding PA from CZ, together with GFP (D) or Rluc (E) expression vectors. Cells expressing GFP were observed using an inverted fluorescence microscope under identical exposure conditions and then assessed by flow cytometry. Cells expressing Rluc were performed by Luciferase assays. (F) qPCR analysis of JAK1 mRNA level in A549 cells transfected with different concentrations of PA plasmids (from 0.5, 1 to 1.5 μg) (n = 4). (G) Immunoblots of HEK293T cells were transfected with indicated plasmids, and the amount of each plasmid was indicated above the lanes (in microgram) (upper). The intensities of the bands on the immunoblots from three independent experiments were quantified and normalized with actin (lower). (H) Immunoblots of HEK293T cells infected with CZ virus at an MOI of 0.1 (left). The intensities of the bands on the immunoblots from three independent experiments were quantified and normalized with actin (right). hpi, h post-infection. (I) Immunoblots of HEK293T cells treated with different concentrations of IFNβ (from 10, 100 to 1000U/ml) (left). The intensities of the bands on the immunoblots from three independent experiments were quantified and normalized with actin (right). Data are presented as the mean ± SD. Statistical significance was determined by unpaired two-tailed Student’s t-test in A–C, E–I. ns P >0.05. (TIF) [file ppat.1011489.s002.tif]

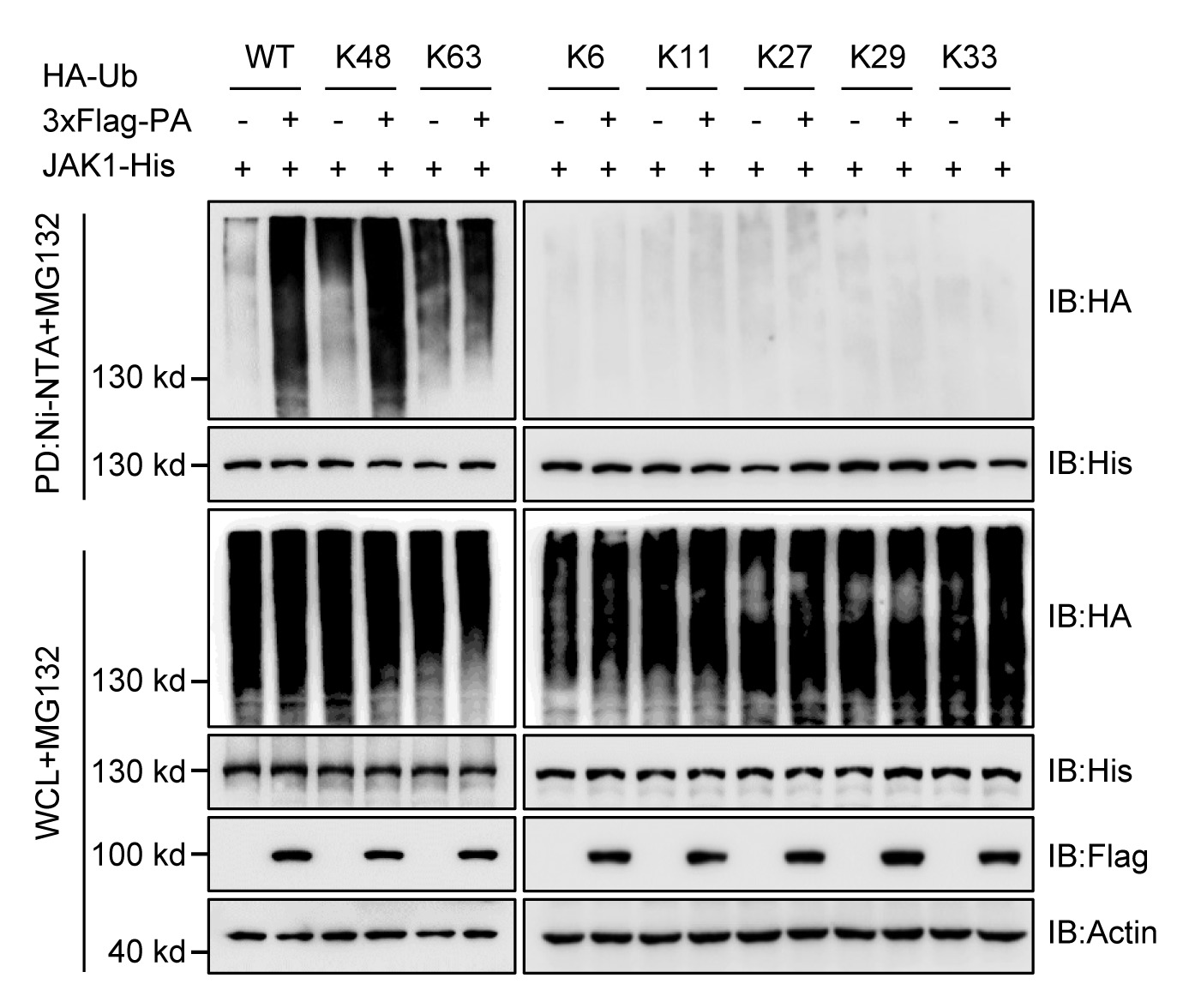

Supplement: S3 Fig — Ni-NTA pull-down analysis of the ubiquitination of JAK1 in HEK293T cells transfected with JAK1, HA-Ub or its mutants [K at indicated residue, and K at other residues were simultaneously mutated to arginines], and PA plasmids and treated with MG132. Data are representative of two independent experiments. (TIF) [file ppat.1011489.s003.tif]

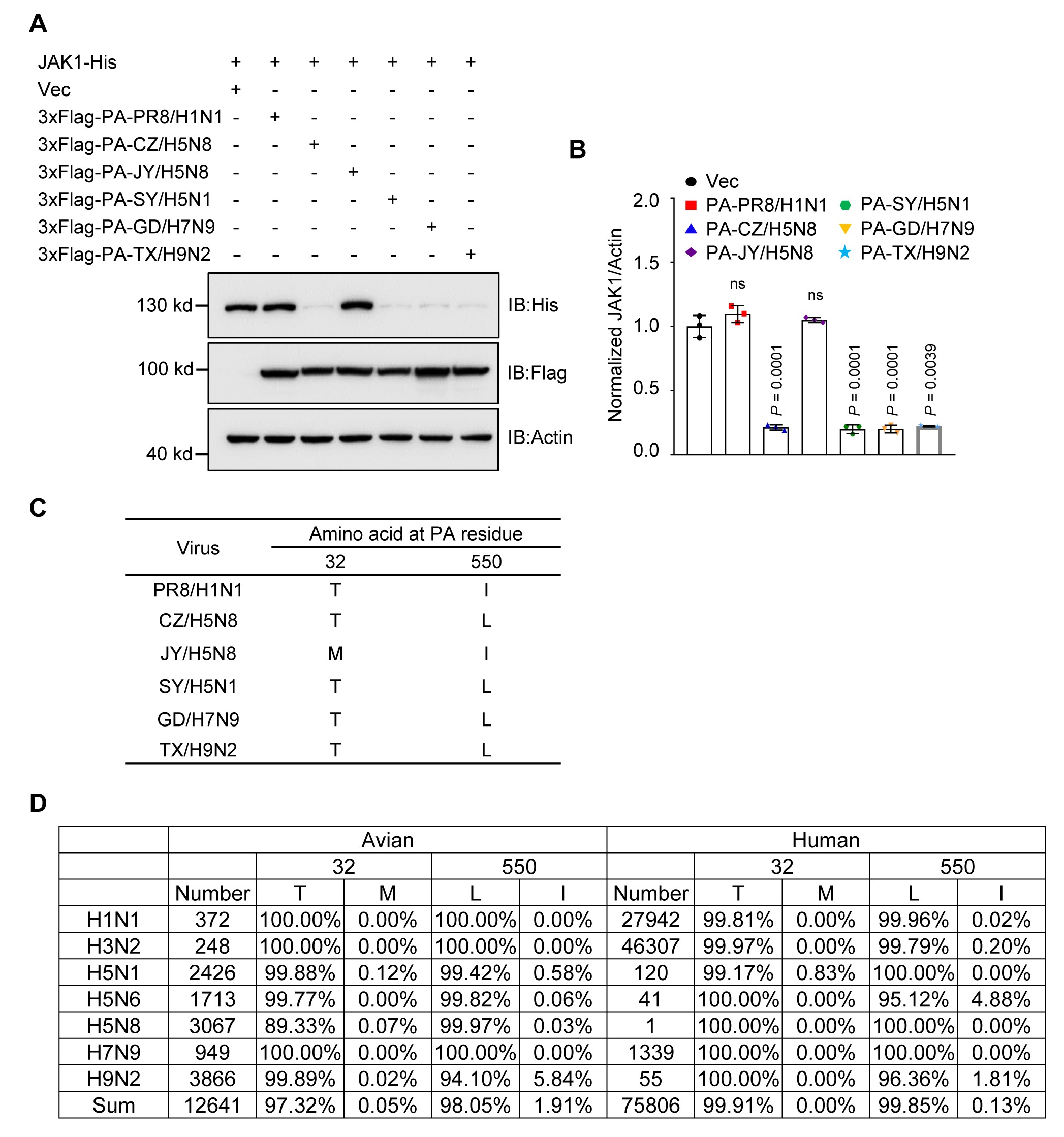

Supplement: S4 Fig — (A) Immunoblots of HEK293T cells transfected with different IAV PA plasmids. (B). Quantification of protein expression on immunoblots (A) and normalized with actin (n = 3). Data are presented as means ± SD and statistical significance was determined by unpaired two-tailed Student’s t-test. (C) The amino acid differences between the PA protein from different IAVs. (D) Frequencies of different residues at PA 32 and 550 positions in avian and human viruses. All sequences were obtained from GISAID database. (TIF) [file ppat.1011489.s004.tif]

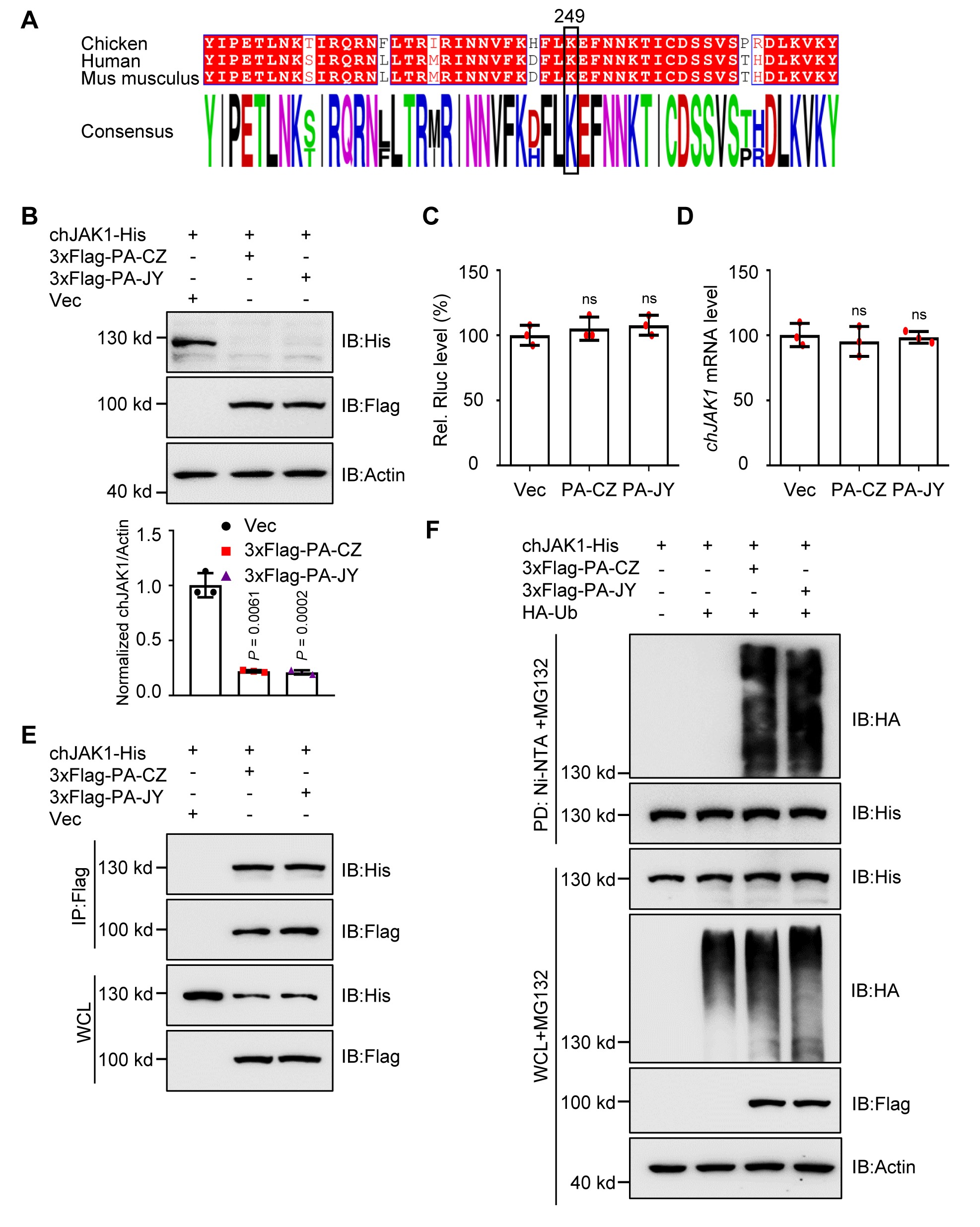

Supplement: S5 Fig — (A) Amino acid alignment of JAK1 from the different species. The black box highlights amino acid 249 corresponding to human JAK1. (B) Immunoblots of HEK293T cells transfected with PA and chJAK1 plasmids (upper). Densitometry analysis of the ratio of JAK1/Actin on immunoblots from three independent experiments (lower). (C) Luciferase assays in DF-1 cells transfected with Rluc plasmid, and PA-CZ or PA-JY plasmid (n = 3). (D) qPCR analysis of chJAK1 mRNA level in DF-1 cells transfected with PA plasmids from CZ and JY. (n = 3). (E) Co-ip analysis of the interaction of PA with chJAK1 in HEK293T cells. (F) Ni-NTA pull-down analysis of the ubiquitination of chJAK1 in HEK293T cells transfected with chJAK1, HA-Ub, and PA plasmids. WCL, whole-cell lysates. Data are presented as means ± SD and statistical significance was determined by unpaired two-tailed Student’s t-test in B–D. ns P >0.05. Data are representative of three independent experiments. (TIF) [file ppat.1011489.s005.tif]

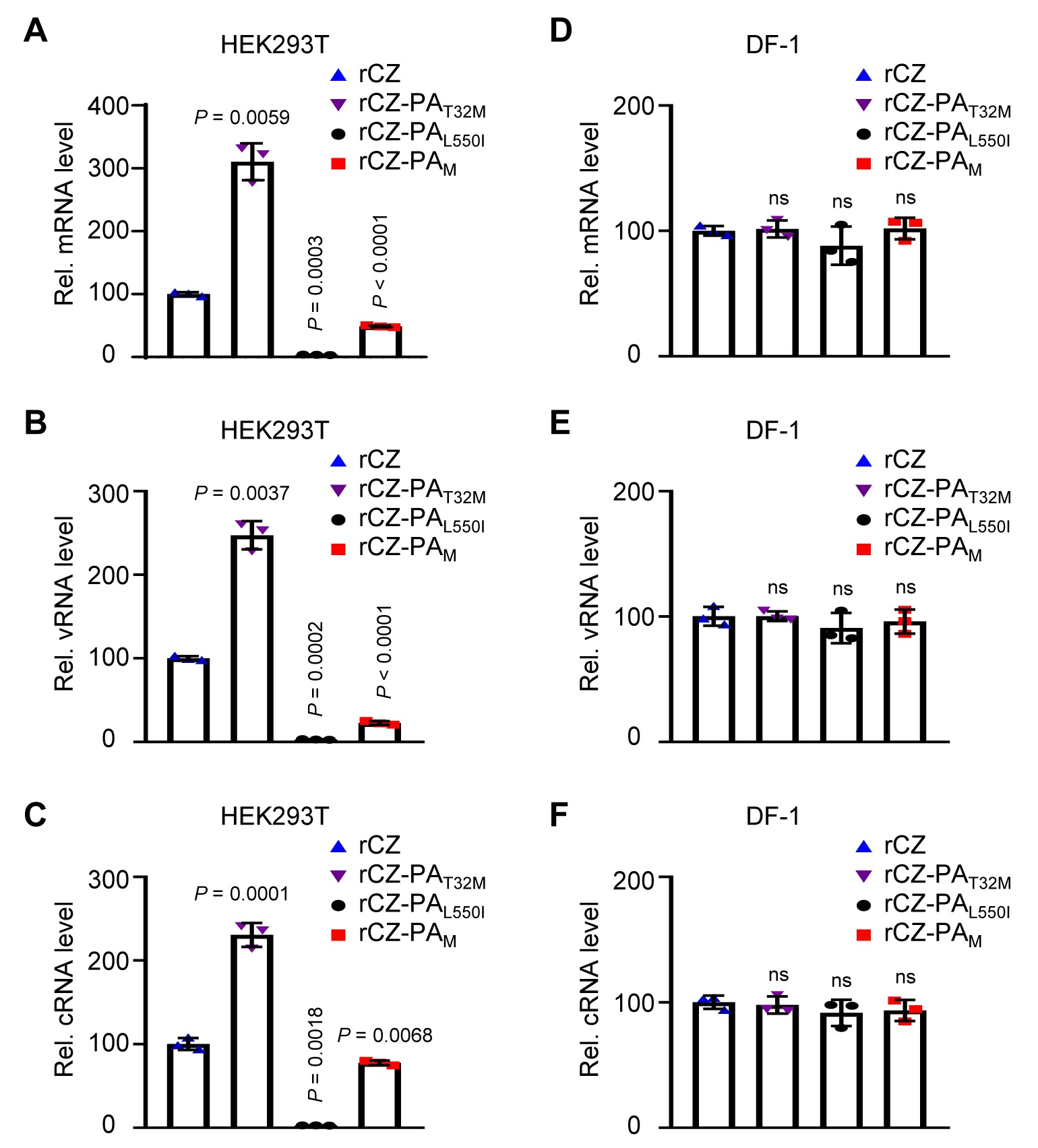

Supplement: S6 Fig — (A–C) HEK293T cells or (D–F) DF-1 cells were infected with rAIVs. Levels of NP genes were estimated by quantitative RT-PCR (n = 3). Data are presented as means ± SD and statistical significance was determined by unpaired two-tailed Student’s t-test. ns P >0.05. (TIF) [file ppat.1011489.s006.tif]

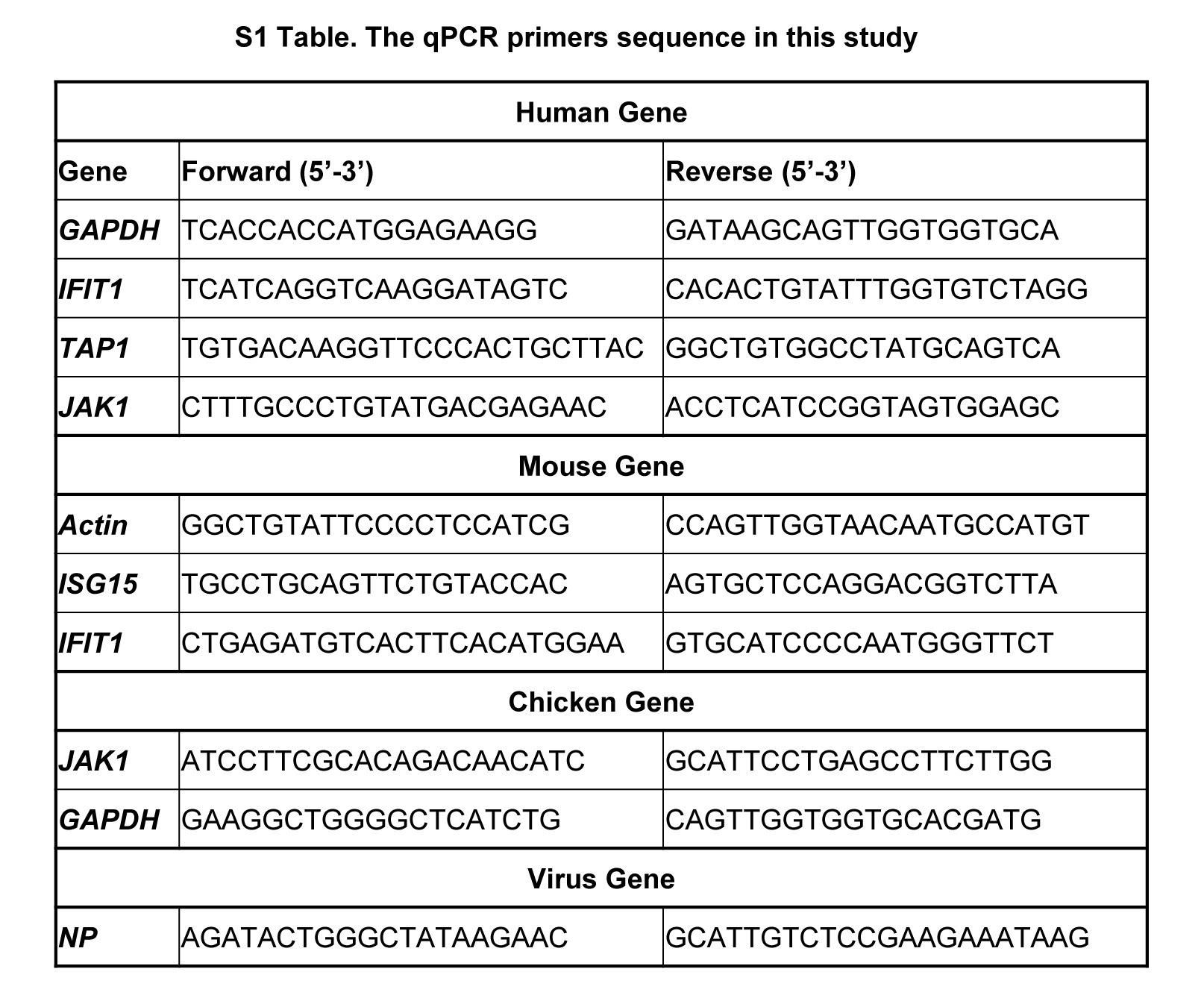

Supplement: S1 Table — (TIF) [file ppat.1011489.s007.tif]
